# Effect of a synthetic hydroxyapatite-based bone grafting material compared to established bone substitute materials on regeneration of critical-size bone defects in the ovine scapula

**DOI:** 10.1093/rb/rbae041

**Published:** 2024-04-24

**Authors:** Jonas Wüster, Norbert Neckel, Florian Sterzik, Li Xiang-Tischhauser, Dirk Barnewitz, Antje Genzel, Steffen Koerdt, Carsten Rendenbach, Christian Müller-Mai, Max Heiland, Susanne Nahles, Christine Knabe

**Affiliations:** Department of Oral and Maxillofacial Surgery, Charité—Universitätsmedizin Berlin, Corporate Member of Freie Universität Berlin, Humboldt-Universität zu Berlin, and Berlin Institute of Health, Berlin, Germany; Department of Oral and Maxillofacial Surgery, Charité—Universitätsmedizin Berlin, Corporate Member of Freie Universität Berlin, Humboldt-Universität zu Berlin, and Berlin Institute of Health, Berlin, Germany; Department of Experimental Orofacial Medicine, Philipps University Marburg, Germany; Department of Experimental Orofacial Medicine, Philipps University Marburg, Germany; Veterinary Research Centre, Bad Langensalza, Germany; Veterinary Research Centre, Bad Langensalza, Germany; Department of Oral and Maxillofacial Surgery, Charité—Universitätsmedizin Berlin, Corporate Member of Freie Universität Berlin, Humboldt-Universität zu Berlin, and Berlin Institute of Health, Berlin, Germany; Department of Oral and Maxillofacial Surgery, Charité—Universitätsmedizin Berlin, Corporate Member of Freie Universität Berlin, Humboldt-Universität zu Berlin, and Berlin Institute of Health, Berlin, Germany; Department of Orthopaedics and Traumatology, Hospital for Special Surgery, Lünen, Germany; Department of Oral and Maxillofacial Surgery, Charité—Universitätsmedizin Berlin, Corporate Member of Freie Universität Berlin, Humboldt-Universität zu Berlin, and Berlin Institute of Health, Berlin, Germany; Department of Oral and Maxillofacial Surgery, Charité—Universitätsmedizin Berlin, Corporate Member of Freie Universität Berlin, Humboldt-Universität zu Berlin, and Berlin Institute of Health, Berlin, Germany; Department of Experimental Orofacial Medicine, Philipps University Marburg, Germany

**Keywords:** osteogenesis, osteogenic markers, bone grafts, TCP, calcium phosphate, hydroxyapatite

## Abstract

Lately, the potential risk of disease transmission due to the use of bovine-derived bone substitutes has become obvious, demonstrating the urgent need for a synthetic grafting material with comparable bioactive behaviour and properties. Therefore, the effect of a synthetic hydroxyapatite (HA) (Osbone^®^) bone grafting material on bone regeneration was evaluated 2 weeks, 1 month, and 3, 6, 12 and 18 months after implantation in critical-size bone defects in the ovine scapula and compared to that of a bovine-derived HA (Bio-Oss^®^) and β-tricalcium phosphate (TCP) (Cerasorb^®^ M). New bone formation and the biodegradability of the bone substitutes were assessed histomorphometrically. Hard tissue histology and immunohistochemical analysis were employed to characterize collagen type I, alkaline phosphatase, osteocalcin, as well as bone sialoprotein expression in the various cell and matrix components of the bone tissue to evaluate the bioactive properties of the bone grafting materials. No inflammatory tissue response was detected with any of the bone substitute materials studied. After 3 and 6 months, β-TCP (Cerasorb^®^ M) showed superior bone formation when compared to both HA-based materials (3 months: β-TCP 55.65 ± 2.03% vs. SHA 49.05 ± 3.84% and BHA 47.59 ± 1.97%; *p *≤* *0.03; 6 months: β-TCP 62.03 ± 1.58%; SHA: 55.83 ± 2.59%; BHA: 53.44 ± 0.78%; *p *≤* *0.04). Further, after 12 and 18 months, a similar degree of bone formation and bone–particle contact was noted for all three bone substitute materials without any significant differences. The synthetic HA supported new bone formation, osteogenic marker expression, matrix mineralization and good bone-bonding behaviour to an equal and even slightly superior degree compared to the bovine-derived HA. As a result, synthetic HA can be regarded as a valuable alternative to the bovine-derived HA without the potential risk of disease transmission.

## Graphical abstract

**Figure:**
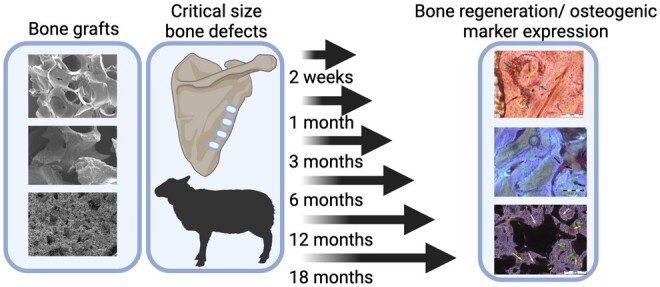

## Introduction

Oral rehabilitation with dental implants often requires bone augmentation prior to or simultaneously with implant placement in order to achieve long-term implant stability and to meet the demands of restoration-driven implant placement [1]. As such, grafting procedures are often needed to reconstruct alveolar defects after tooth loss and to generate sufficient bone volume in both vertical and horizontal dimensions. At the same time, long-term stable contour augmentation is achieved, which is important for obtaining favourable aesthetic outcomes, optimal peri-implant soft tissue conditions and oral hygiene maintenance [1]. In this context, autologous bone grafts remain the ‘gold standard’ in oral and maxillofacial surgery, but over the last two decades, bone graft substitutes have opened new possibilities for bone repair and reconstruction [2–4], even with critical-size bone defects (CSBDs).

It is essential that an optimal bone graft substitute possesses excellent osteoconductive, osteogenic and bioactive properties and thereby enhances new bone formation [5–7]. Furthermore, the bone grafting material should ideally degrade in the newly formed bone within an adequate timeframe to allow restoration of the original bone microarchitecture and optimal osseointegration of dental implants [7, 8]. At the same time, the degradation products need to be excreted via physiological processes with no adverse effects on surrounding tissues during the resorption process, but rather enhance bone tissue formation. Allogenic, xenogenic and synthetic bone grafting materials as well as combination products have been proposed as alternatives to autogenous bone grafts. However, there is still no consensus in the scientific literature as to which bone grafting material is best suited for various indications to render results equal to those with autogenous bone grafts [9, 10]. Frequently used bone grafting materials are bioactive calcium phosphate (CaP), ceramics and glasses, which are regarded as excellent bone substitutes for bone augmentation due to their excellent bone-bonding ability and their enhancement effect on bone formation [7, 11, 12]. Within this group, β-tricalcium phosphate (β-TCP) [13–15], hydroxyapatite (HA) [16–18] as well as biphasic TCP/HA bone substitutes [19, 20] are known for their favourable osteoconductive properties and biocompatibility [13–17]. While HA has been considered advantageous due to its composition being structurally and functionally similar to the mineral composition of natural bone tissue [17, 18, 21], the use of β-TCP has been promoted due to its higher biodegradability [7, 22], which may, however, impair the volume stability of the augmented tissue [23]. Moreover, β-TCP has excellent osteoconductive properties, which support the adhesion and proliferation of osteoblasts [24–26] and stimulate new bone formation [24, 27]. The balance between rapid bone formation and concurrent biodegradation is of particular interest in alveolar ridge augmentation and subsequent dental implant placement since osseointegration and a preferably high bone–implant contact requires the formation of functional bone tissue at the dental implant surface [14]. The use of HA-based bone substitutes reaches back to the 1980s [28], since its mineral composition is most similar to that of natural bone, and it is still widely used today [21]. Compared to TCP, HA is mechanically more stable and less soluble [29, 30]; both of these factors directly affect the biodegradation and volume stability of areas regenerated with bone substitutes [23]. As a result, a combination of both types of bone grafting materials has been proposed for alveolar ridge augmentation using the guided bone regeneration (GBR) technique and a staged approach. Whereas a CaP material with higher resorbability such as TCP is used for the central area in which the implants are to be placed, the outer contour is restored with a CaP material with lower resorbability (such as HA) to achieve high volume stability of the convex outer contour, which is of particular importance in the aesthetic zone [31].

Despite the major advantages of synthetic bone graft substitutes, i.e. avoiding the risk of donor site morbidity [32], various limitations exist for each group. Although some studies have demonstrated allogenic bone blocks to be a reliable alternative to autogenous bone for certain indications due to their excellent osteoconductive behaviour [33, 34], other studies have revealed a higher complication rate compared to autogenous bone grafts [35]. The recent tuberculosis outbreak resulting from the use of bone allografts has emphasized the inherent risk of disease transmission, which cannot be fully ruled out with these materials, and the great need for synthetic bone substitute materials with comparable bone regenerative properties [36]. Among xenogenic bone substitutes, bovine grafting materials are probably the most studied subgroup. They appear to be a reliable bone graft substitute for certain indications such as socket preservation and can even render the same clinical outcomes as autologous bone [37–41]. Despite the clinical success of bovine-derived bone grafting materials demonstrated by some groups, several studies have reported complications, such as initial minor inflammation [42]. In addition, Kim et al. even suggested abolishing the use of bovine-derived bone substitutes due to the risk of disease transmission [43–45]. All these particular limitations demonstrate the urgent need for a synthetic grafting material with comparable bioactive behaviour and properties. This led to the development of novel highly porous synthetic HA granules with an open interconnected porosity, which was designed for high osteoconductivity and bioactivity.

Consequently, the current study aimed at testing the hypothesis that the novel synthetic HA granules (Osbone^®^, Curasan AG, Kleinostheim, Germany) would possess a bone regenerative capacity equal to that of the bovine-derived HA granules (Bio-Oss^®^, Geistlich Pharma AG, Wolhusen, Schweiz). To this end, the effect of the synthetic HA granules (S-HA) on osteogenic marker expression and bone regeneration of CSBDs created in sheep scapulae was analyzed after 2 weeks, 1 month, and 3, 6, 12 and 18 months of implantation and compared to that of the bovine-derived HA granules (B-HA) with clinically established alloplastic β-TCP granules (Cerasorb^®^ M (Curasan AG, Kleinostheim, Germany)) serving as a reference, whose excellent osteogenic properties have been widely documented.

## Materials and methods

### Test materials

The bone grafting materials utilized in this study were synthetic powdered ≥99% pure phase HA granules (Osbone^®^ (Curasan AG, Kleinostheim, Germany)) with a particle size of 1000–2000 µm; a pure HA of bovine origin (a well-known non-resorbable bone void filler) with a granule size of 1000–2000 µm (Bio-Oss^®^ (Geistlich, Wolhusen, Switzerland)); and a ≥ 99% pure phase β-TCP, denominated Cerasorb^®^ M (Curasan AG, Kleinostheim, Germany), which served as reference (Table 1). All three materials received the CE mark, were cleared by the Food and Drug Administration (FDA) and were sterilized by the respective supplier using gamma irradiation at 25 kGy. An overview of the material properties is given in Table 1.

**Table 1.** Overview of the three tested materials: β-TCP (Cerasorb^®^ M), synthetic HA (Osbone^®^) and bovine HA (Bio-Oss^®^) [14, 46, 47]

Material	Composition	Description	Granule size	Pore size	Porosity
TCP (Cerasorb^®^ M)	Pure phase b-tricalcium phosphate β-TCP, Ca_3_(PO4)_2_	Porous	1000–2000 µm	0.1–500 µm	65%
Synthetic HA (Osbone^®^)	Pure phase HA Ca_10_(PO_4_)_6_(OH)_2_	Open-cellular	1000–2000 µm	250–450 µm	80 ± 5%
Bovine HA (Bio-Oss^®^)	Pure HA of bovine origin Ca_10_(PO_4_)_6_(OH)_2_	Porous	1000–2000 µm	150–1000 µm	75–80%

Osbone^®^ granules were manufactured by first fabricating parts with a sponge-like morphology utilizing a replica technique, for which combustible polyurethane foam templates were utilized [14, 30]. In short, a homogenous water-based slurry was prepared with the HA powder material, followed by coating a cellular polyurethane foam with the slurry so as to create an open cellular structure. After drying at 50°C, the coated foam was sintered at 1000°C, which involved pyrolysis of the foam. After sintering, the obtained highly porous parts were crushed (Pulverisette, Fritsch, Germany) to fabricate the desired granule size of 1000–2000 µm. Finally, sieving was applied for granule size refinement. TCP granules were manufactured by mixing TCP with powder ammonium hydrogen carbonate, which served as porogen. This was followed by isostatic pressing at 200 MPa, sublimation of the porogen at 80°C, and finally sintering at 1000°C, as described elsewhere [14, 30]. Bio-Oss granules were manufactured from bovine bone involving deproteinization, as described elsewhere [47, 48].

### Material characterization

The surface morphology of the three bone grafting materials studied was visualized by scanning electron microscopy (SEM; Type Amray 181). Micro-computed tomography (μ-CT40, Scanco Medical, Switzerland) was applied to measure the porosity and strut size of the test materials in triplicate (*n* = 3). The physicochemical properties of the materials were analyzed utilizing Fourier transform infrared spectroscopy (FTIR; Alpha FTIR, Bruker Tensor 37, Bruker, Germany) and X-ray diffraction (XRD) analysis, as described previously [14, 30]. In brief, the ATR (attenuated total reflection) technique was applied when characterizing the chemical bonds present in the samples by FTIR. Analyses were carried out at room temperature in the range of 30–3600 cm^−1^ with a resolution of 4 cm^−1^. Each FTIR measurement involved scanning the specimens 16 times, and the spectrum acquired was the average of these 16 scans. For XRD analysis (XPERT-PRO, Panalytical, Germany), a Cu Kα radiation source with a wavelength of 1.79 Å at a voltage of 35 kV and a current of 35 mA was utilized. Patterns were obtained for 2θ values of 10–110° in a continuous mode and with a step size of 0.026° (2θ), as described elsewhere.

### Animal surgery

Animal ethics approval was granted by the Thuringian Veterinary Council (14-001/09) in accordance with the German and European Animal Welfare guidelines. Animal experiments were conducted at a veterinary hospital and research centre (FZMB GmbH, Bad Langensalza, Thuringia, Germany). Surgery was performed on 36 merino sheep, 24 months in age (six animals at each time point: 2 weeks and 1 month, 3, 6, 12 and 18 months). Prior to surgery, all animals received a standard diet of hay and water *ad libitum*. The surgery was performed according to a previously established study protocol that showed no decrease in the stability of the scapula and no fractures or other complications [14]. Intravenous induction of the anaesthesia entailed the administration of 0.2 mg kg^−1^ diazepam (Ratiopharm GmbH, Germany) and 4 mg kg^−1^ ketamine (Inresa GmbH, Germany), followed by tracheal intubation and maintenance of the anaesthesia with 1–1.5 vol% isoflurane in oxygen. During surgery, systemic antibiotic prophylaxis (0.06 ml kg^1^ Veracin-Compositum, Albrecht GmbH, Germany) and analgesic treatment (Carprofen, 4 mg kg^−1^) were administered intravenously. Through a longitudinal skin incision over the left scapula, the periosteum was exposed and incised in a longitudinal manner (∼12 cm). Subsequently, the preparation of a periosteal flap and the creation of the CSBDs followed. As described in our previous study [14], CSBDs consisted of holes 8 mm in diameter and 8 mm in depth, which were prepared in the cancellous section of the inferior margin of the left scapula using a trephine bur under copious saline irrigation. Four CSBDs were created in each scapula, at least 10 mm of space was maintained between the defects, and the ends of the outer holes were marked with titanium pins to facilitate the relocation of the defects at implant retrieval. The three test materials, with a particle size of 1000–2000 µm, were implanted randomly in each defect, while empty defects (ED) served as negative controls. The test materials were examined after implantation at 2 weeks, 1 month, and 3, 6, 12 and 18 months. For each time point, a group of 6 animals was selected, and each animal received all three test materials and an ED. Wound closure was performed applying a multilayer technique, using resorbable 4-0 sutures (Monocryl, Johnson & Johnson, New Brunswick, USA) for the muscle tissue and resorbable 5–0 sutures (Vicryl, Johnson & Johnson, New Brunswick, USA) for the skin. At the predefined time points, the selected animals were sacrificed, and the bone tissue blocks with the implanted materials and the surrounding tissue were obtained and fixed in HistoCHOICE^TM^ (AMRESCO, Solon, USA) solution for 7 days at room temperature.

### Histology and immunohistochemistry

All bone samples were processed as described in previous studies [14, 48, 49], allowing performing immunohistochemical staining and analysis on undecalcified hard tissue sections. After fixing the tissue blocks in the ethanol-based fixative HistoCHOICE™ (AMRESCO, Solon, USA), samples were embedded in a resin composed of pure methyl methacrylate (PMMA), *n*-butyl-methacrylate (BMA), benzoyl peroxide (BPO) (catalyst), polyethylene glycol 400 and 1.5 ml *N*,*N*-dimethyl-*p*-toluidine (VWR, Darmstadt, Germany). Both the fixative as well as the specific resin chosen facilitated maintaining the antigenicity of the tissue in contrast to conventional formaldehyde fixation and PMMA embedding. The polymerized blocks were glued to acid-resistant acrylic slides (Plexiglas slides #404150/GLS, Walter Messner GmbH, Germany) utilizing an epoxy resin–based two-component adhesive (UHU Inc., Bühl, Germany). Perpendicularly to the long axis of the cylindrical defect, 50-µm sections were cut with a Leitz 1600 sawing microtome (Leitz, Wetzlar, Germany) and then ground and polished with 1200- and 400-grit wet silicon carbide grinder (Exakt grinding system 400 CS, Exakt Corporation Norderstedt, Germany). In order to facilitate immunohistochemical staining, deacrylation of sections was performed by immersion in toluene, xylene and acetone, which resulted in the removal of the resin from the sections. The special glue and acrylic slides outlined above were chosen so as to withstand the deacrylation procedure, which was followed by incubation with 2% bovine serum albumin (BSA) (Sigma, St Louis, USA) in DAKO antibody diluent (DAKO Corporation, Glostrup, Denmark) for 20 min and then endogenous peroxidase in Peroxidase Enzyme Blocking Solution (DAKO Corporation, Glostrup, Denmark) for 10 minutes to block the non-specific binding site. Immunohistochemical staining was performed using primary mouse monoclonal antibodies specific to alkaline phosphatase (ALP) (ALO, Sigma-Aldrich Corp., St Louis, USA) and osteocalcin (OC) (Abcam Ltd, Cambridge, UK), and rabbit polyclonal IgG antibodies against bone sialoprotein (BSP, LF-84, National Institute of Health, Bethesda, USA) and type I collagen (Col I, LF-39, National Institute of Health, Bethesda, USA). Non-immunized mouse and rabbit IgG PP54 and PP64 (PP54 and PP64, Merck KGaA Millipore, Billerica, USA) were used as negative controls, and Mayer’s haematoxylin was used as a counterstain. Coverslips were mounted using Kaiser’s glycerol gelatine (Merck AK, Darmstadt, Germany). The AEC system leads to a bright red and/or brownish colour, whereas counterstaining with haematoxylin yields a violet/bluish colour, enabling the distinction between positively stained cells/matrix components and cells/matrix components with negative immunostaining [14, 48–50]. Additional sections were stained with Giemsa stain and von Kossa stain, modified according to Gross and Strunz [51]. Furthermore, tartrate-resistant acid phosphatase staining was performed using enzyme histochemistry and a respective kit (Sigma-Aldrich Corp., St Louis, Missouri, USA) based on the method by Goldberg & Barka 1962, which detects and identifies cells with osteoclastic activity.

Histomorphometric analysis was performed on a pair of immunohistochemically stained sections 150-µm apart as established in previous studies [14, 49, 50]. To this end, per material and time point, six pairs of stained sections were analyzed after generating MIA (multi-image alignment) panoramic images utilizing a light microscope (Olympus, Germany) using 10× and 20× magnification in combination with a digital camera (Colourview III), and SIS Analysis™ software (Olympus, Germany), which was also employed for carrying out the histomorphometric measurements. The region of interest was defined within the cross-sectional circular area of the defects, 8 mm in diameter. The surface area of newly formed bone and the surface area of the graft material within the bone defect was measured in mm^3^. Then, the area fraction of the newly formed bone and residual graft material was calculated as a percentage of the total. In addition, the bone-biomaterial particle contact was determined by measuring the length of the bone-biomaterial granule contact and expressing it as percentage of the total tissue granule contact. The data obtained from each pair of sections were averaged.

As described in previous publications by our group, semi-quantitative analysis of the immunohistochemically stained sections was performed by two experienced investigators with both investigators blinded to the staining using a light microscope [14, 49, 50, 52]. Tissue components for examination of antibody decoration of cellular (fibroblasts, osteoblasts and osteocytes) as well as matrix components (osteoid seams, trabecular bone and fibrous matrices) were identified on morphological grounds. To quantify the amount of staining, a scoring system was employed, whereby the intensity of staining was assessed as mild (+), moderate (++) and strong (+++) staining, and whether the staining was localized (+) or generalized (**+**). This methodology and color development has been described in detail elsewhere [15, 48, 49] including negative control sections stained with non-immunized mouse and rabbit IgG. As such, a numerical score of 5, 3 and 2 corresponded to generalized strong, moderate or mild staining, whereas a score of 4, 2 and 1 indicated strong, moderate or mild staining in localized areas. A score of (0) was used for no staining. The average score of the 6 sections per osteogenic marker was calculated for each cell and matrix component. An average score of 3.5–5 was evaluated as strong expression of a respective marker in a given cellular or matrix component, and an average score of (2.3–3.4), (1–2.2), or (0.1–0.9) was assessed as moderate, mild or minimal expression [48]. The beauty of this technique is that osteoblasts at their different stages of differentiation can be visualized at the bone-bioceramic interface as well as in the pores of the degrading bioactive ceramic granules, thereby allowing to examine the actual bioactive properties of these bioactive ceramic bone grafting materials *in vivo*. The same is true for areas of progressing bone matrix maturation and mineralization [14, 49, 50, 52].

### Statistical analysis

For all data, mean ± SD was calculated. Statistical analysis such as the Kruskal–Wallis test for multiple comparisons between groups and the Mann–Whitney *U* test in combination with Bonferroni adjustment was performed. Calculations were performed using the statistical software SPSS Statistics 16.0 (SPSS Inc., Chicago, Illinois, USA). The probability level for statistical significance was set at *p *≤* *0.05.

## Results

### Material characterization

#### SEM analysis

**Figure 1.**
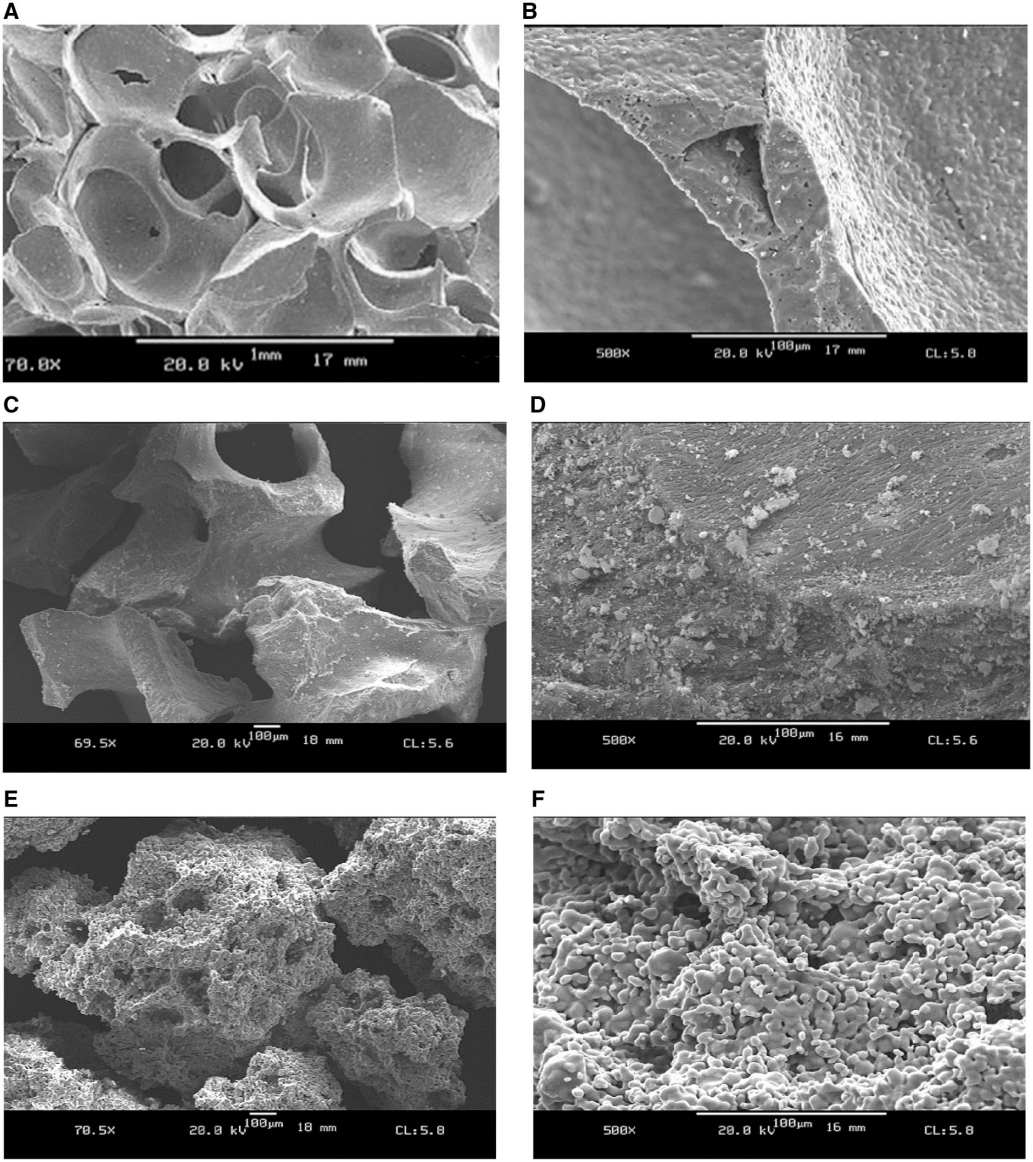
Scanning electron micrographs of (**A**, **B**) SHA (Osbone^®^) granules (A: bar = 1 mm, B: bar = 100 µm); (**C**, **D**) BHA (Bio-Oss^®^) granules (C, D: bar = 100 µm); (**E**, **F**) TCP (Cerasorb^®^ M) granules (E, F: bar = 100 µm).

The morphology of the different materials studied is depicted in Figure 1. SEM analysis showed that as a result of applying the replica technique (Figure 1A and B), the SHA (Osbone^®^) granules displayed an open cellular and interconnected microarchitecture resembling the microarchitecture of cancellous bone. The surface morphology and microarchitecture of the BHA (Bio-Oss^®^) particles reflected the cancellous morphology of the bovine bone utilized for their fabrication (Figure 1C and D). Due to the use of the porogen, the overall microarchitecture of the TCP granules (Cerasorb^®^ M) differed from that of the SHA and BHA granules, with the TCP granules displaying a more irregular microarchitecture (Figure 1E and F).

#### Micro-CT analysis and pore size distribution

**Figure 2.**
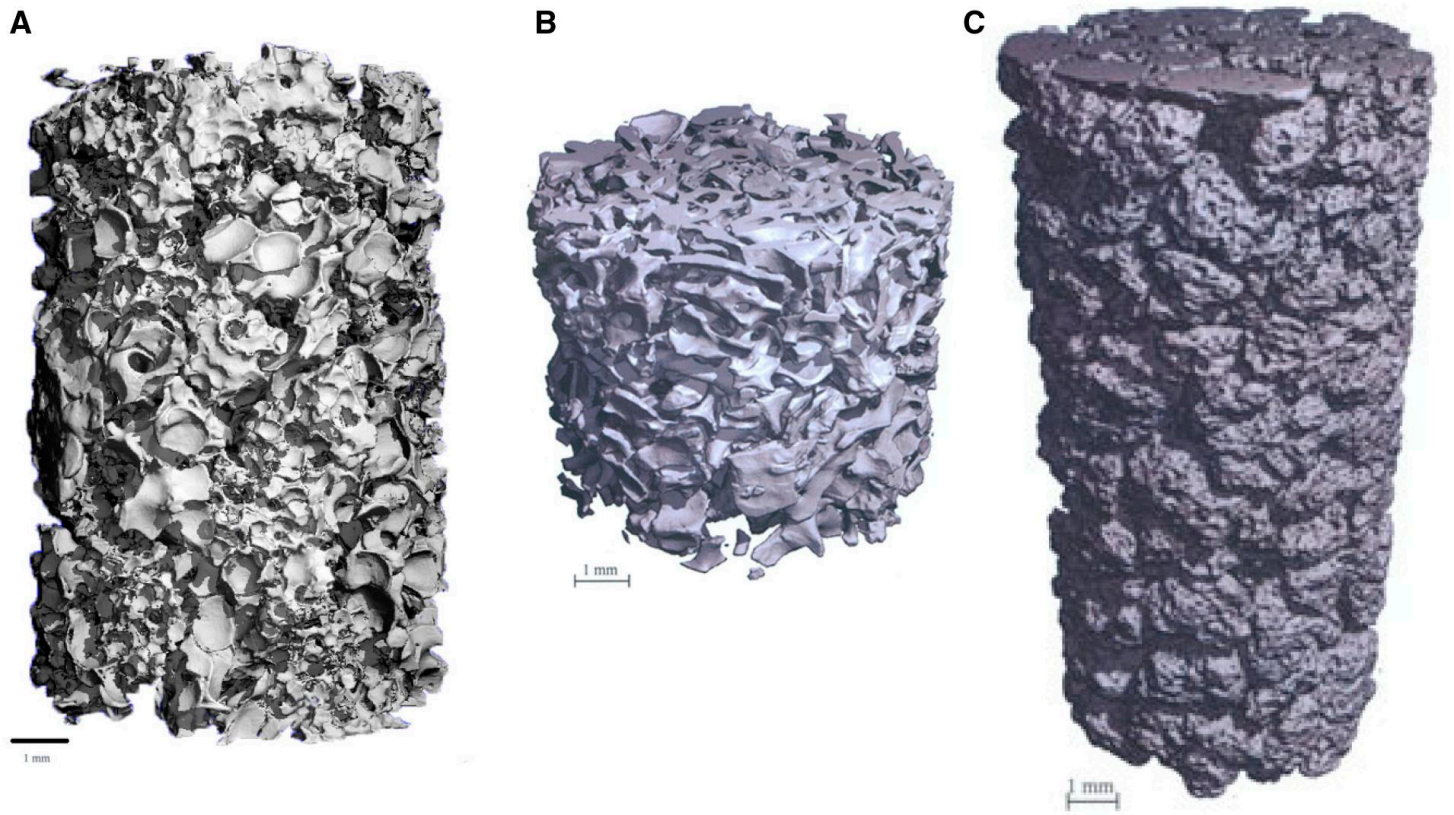
3D μCT Images of vials filled with tightly packed (**A**) SHA (Osbone^®^), (**B**) BHA (Bio-Oss^®^) and (**C**) β-TCP (Cerasorb^®^ M) granules; bar = 1 mm.

**Figure 3.**
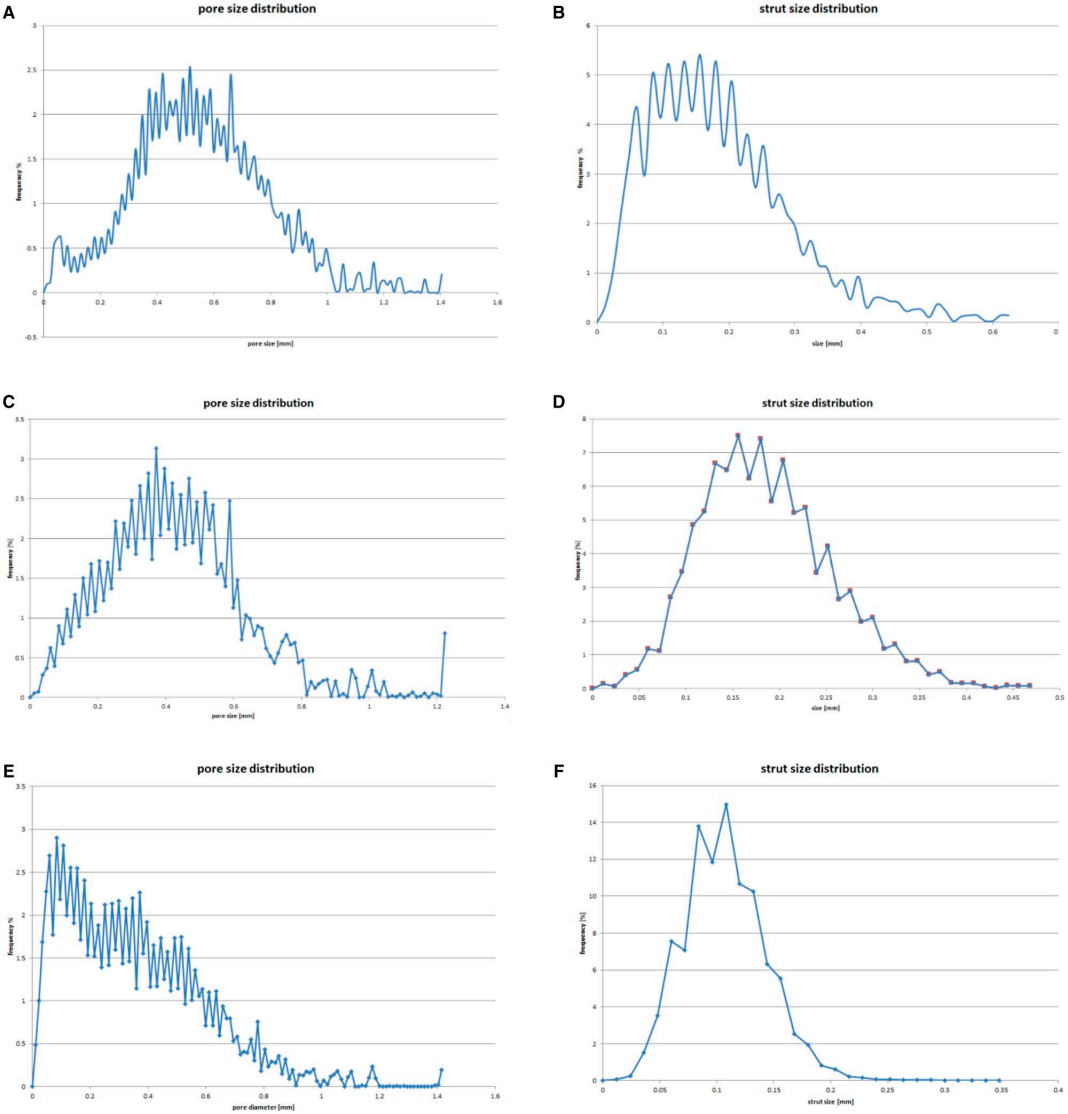
Results of the μCT analysis: pore and strut size distribution in (**A**, **B**) SHA (Osbone^®^), (**C**, **D**) BHA (Bio-Oss^®^) and (**E**, **F**) TCP (Cerasorb^®^ M) granules.

The performed µ-CT analysis enabled three-dimensional visualization of the different bone substitutes, i.e. SHA (Osbone^®^) (Figure 2A), BHA (Bio-Oss^®^) (Figure 2B) and TCP (Cerasorb^®^ M) (Figure 2C). µ-CT analysis furthermore facilitated characterizing the pore size and strut size distribution for SHA (Figure 3A and B), BHA (Figure 3C and D) and TCP (Figure 3E and F). The results of the µ-CT analysis corroborated the findings of the SEM analysis. SHA and BHA displayed a more regular and open microarchitecture than TCP. SHA displayed a mean pore size of 549 ± 235 µm and a mean strut size of 186 ± 107 µm, BHA a mean pore size of 429 ± 209 µm and a mean strut size of 187 ± 70 µm, and TCP a mean pore size of 353 ± 247 µm and a mean strut size of 107 ± 36 µm.

#### XRD analysis

**Figure 4.**
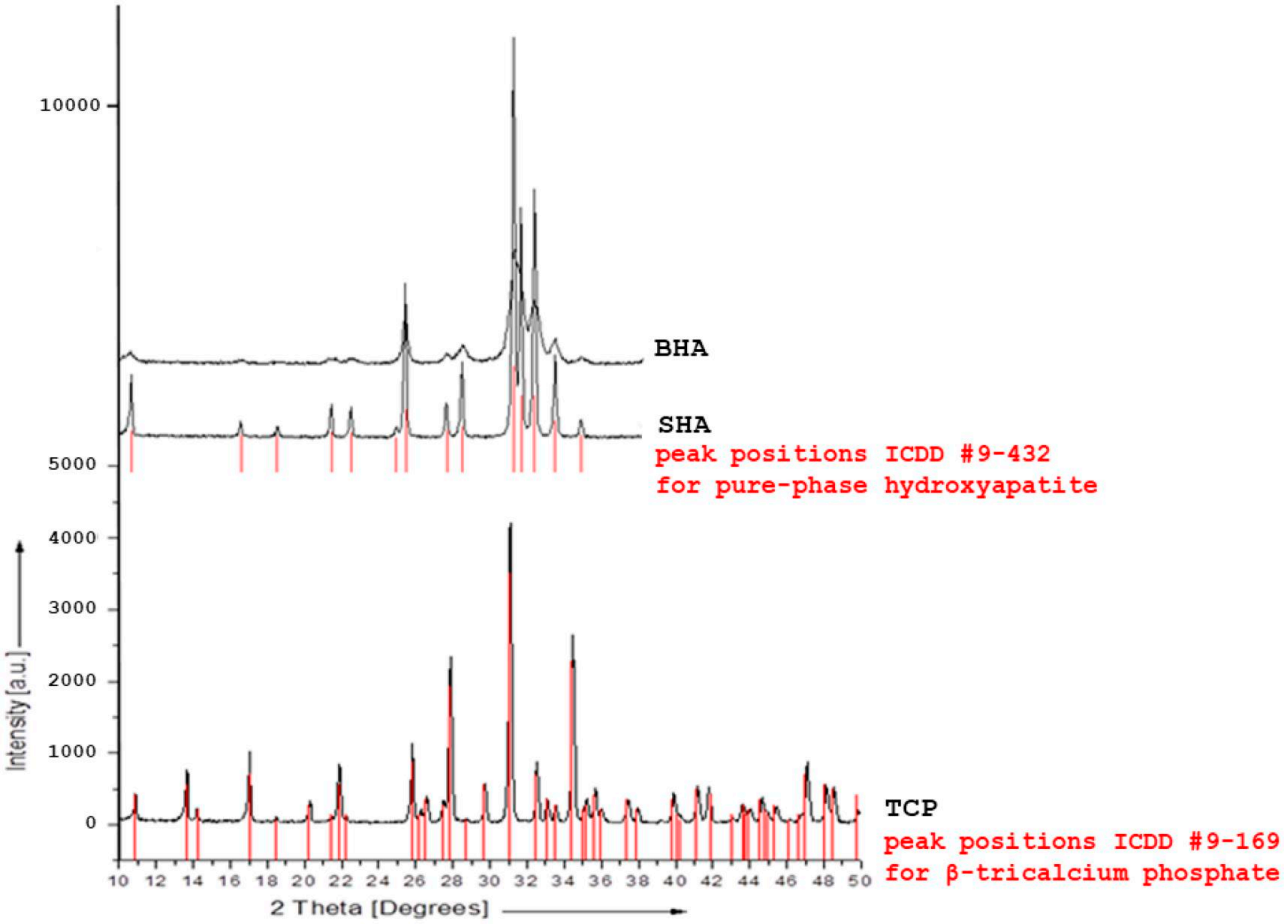
X-ray powder diffractograms of SHA (pure-phase HA Osbone^®^), BHA (Bio-Oss^®^) and TCP (pure-phase β-TCP Cerasorb^®^ M). The red lines indicate the peak positions of the reference files ICDD #9-432 for pure-phase HA and ICDD #9-169 for β-TCP. Excellent agreement of the measured peaks for SHA and TCP with those of the reference substance is visible. In addition, the absence of other foreign peaks or reflections, which might indicate phase impurities, is demonstrated.

The XRD spectra are shown in Figure 4. The XRD pattern of SHA was in agreement with the pattern of HA with reference ICDD 9-432, and that of BHA (Bio-Oss^®^) matched that of calcium*-*deficient carbonate apatite with reference ICDD 9-432 but with broader peaks, which is indicative of a more amorphous character. The XRD pattern of TCP (Cerasorb^®^ M) corresponded to that of β-TCP (ICDD Ref. 9–169). As such, the main peaks for SHA (Osbone^®^) were located at 2θ values around 31.08 and 33.47, and for BHA, around 31.95, 32.34 and 33.25. TCP displayed these peaks at 2θ values around 31.07 and 35.01. The narrow shape of the peaks for SHA and TCP reflects the high crystallinity of the materials. Taken together, the XRD analysis verified the crystalline phases present in the different bone substitutes.

#### FTIR analysis

**Figure 5.**
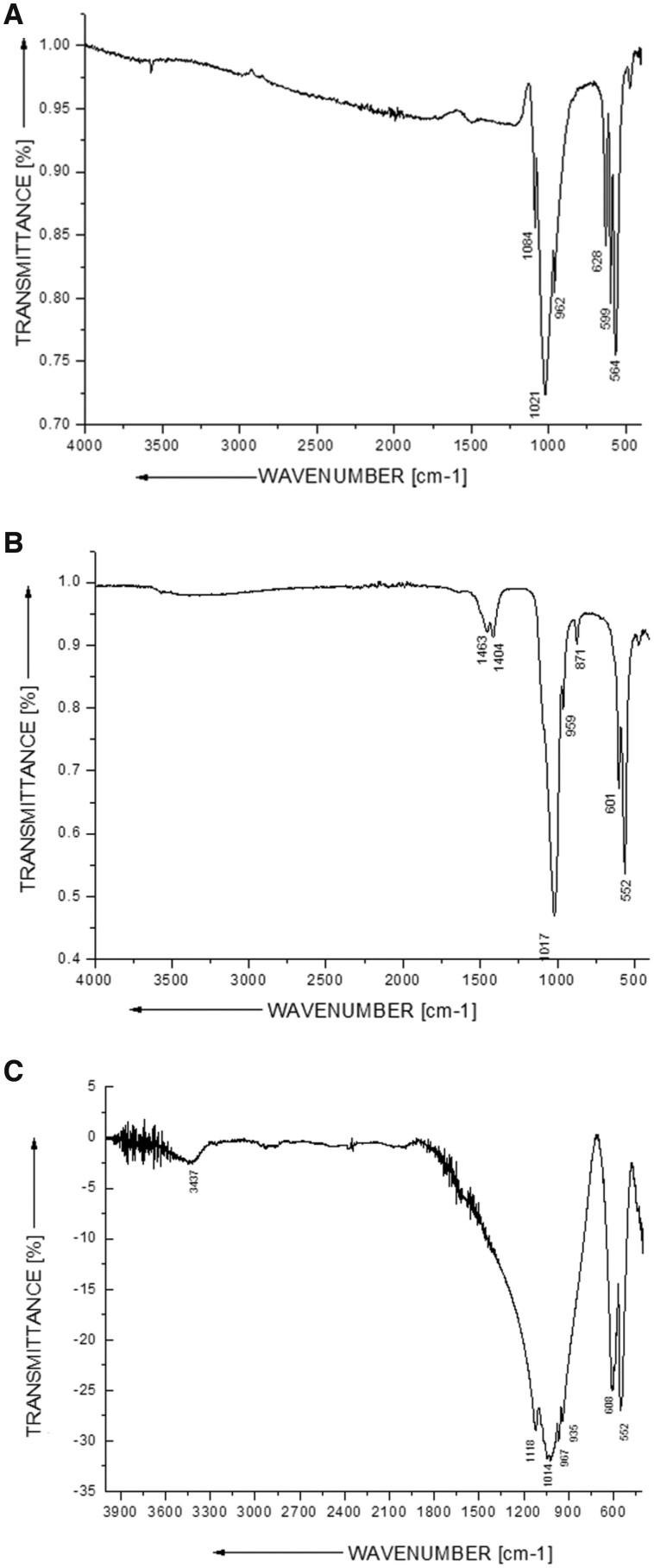
Results of the FTIR analyses: FTIR spectra of (**A**) SHA (Osbone^®^), (**B**) BHA (Bio-Oss^®^) and (**C**) TCP (Cerasorb^®^ M).

FTIR spectra are depicted in Figure 5. The main bands detected were 1087, 1021, 960, 628, 598 and 564 cm^−1^ for SHA (Figure 5A); 1021, 962, 872, 600 and 561 cm^−1^ for BHA (Figure 5B); and 1116, 1002, 967, 941, 601, 589 and 543 cm^−1^ for TCP (Cerasorb^®^ M) (Figure 5C). The corresponding vibrational mode or group is indicated in Supplementary Table S1. These bands corresponded to the ν1, ν2 and ν3 (P-O); ν4 (P-O-P); ν3 (${\text{CO}}_{3}^{2-}$); and νL (OH^-^) groups.

### Clinical findings

In all animals, uneventful healing with no signs of clinical inflammation was observed.

### Histologic and histomorphometric findings and immunohistochemical analysis

**Figure 6.**
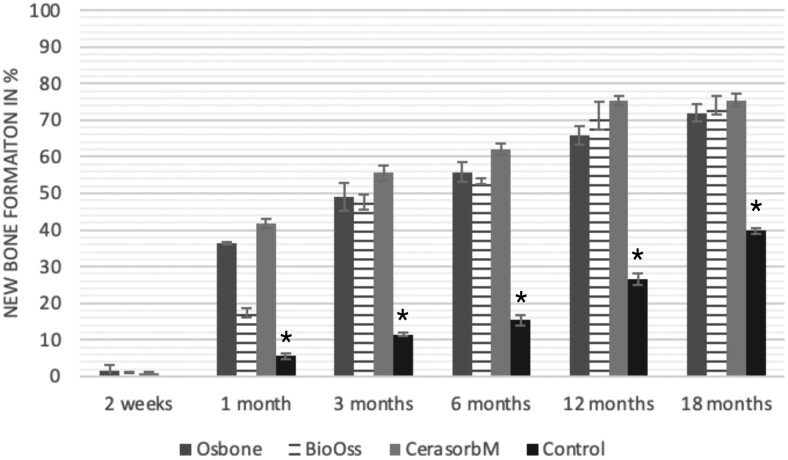
Histograms depicting the results of the histomorphometric analyses. New bone formation of Osbone^®^ (synthetic HA), Bio-Oss^®^ (bovine HA), Cerasorb^®^ M (β-TCP) and the control. Asterisks indicate statistical significance between the empty control and Osbone^®^ (synthetic HA), Bio-Oss^®^ (bovine HA), and Cerasorb^®^ M (β-TCP). All values are mean ± standard deviation of six measurements.

**Figure 7.**
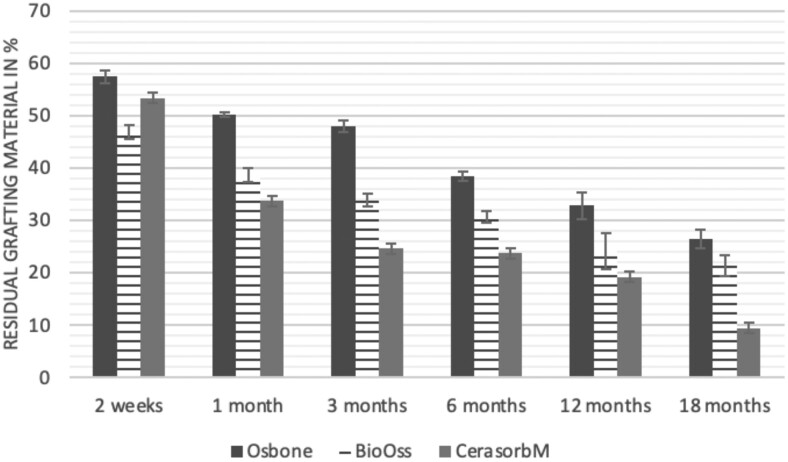
Histograms depicting the results of the histomorphometric analyses. Residual bone grafting material within the defect for Osbone^®^ (synthetic HA), Bio-Oss^®^ (bovine HA) and Cerasorb^®^ M (β-TCP) augmented defects. All values are mean ± standard deviation of six measurements.

**Figure 8.**
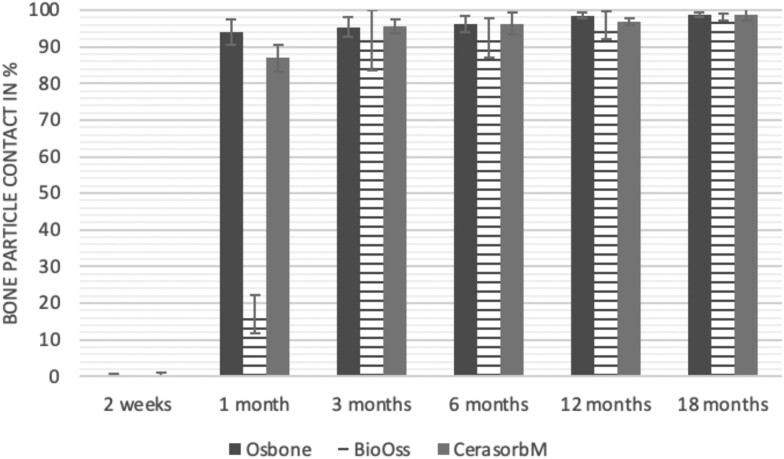
Histograms depicting the results of the histomorphometric analyses. Bone–particle contact for defects augmented with Osbone^®^ (synthetic HA), Bio-Oss^®^ (bovine HA) and Cerasorb^®^ M (β-TCP). All values are mean ± standard deviation of the six measurements.

The results of the histomorphometric analysis are illustrated in Figures 6–8. Examination of osteogenic marker expression in the cells and tissue components formed in the defect area was performed as described, and the results are illustrated in the line charts given in Tables 2–5 (Additional tables with the mean values ± SD are available in the supplementary materials section; Supplementary Tables S2–S5). During the 18-month observation period, no histological signs of inflammation were detected in any group. Representative histomicrographs for all experimental groups are depicted in Tables 6–8.

**Table 2.** Line graphs depicting the mean values of the scores of osteocalcin in the various cell ((A) osteoblasts, (B) osteocytes, (C) fibroblastic cells) and matrix components ((D) fibrous matrix, (E) bone matrix, (F) osteoid) at the various time points^a^

	Figure
(A)	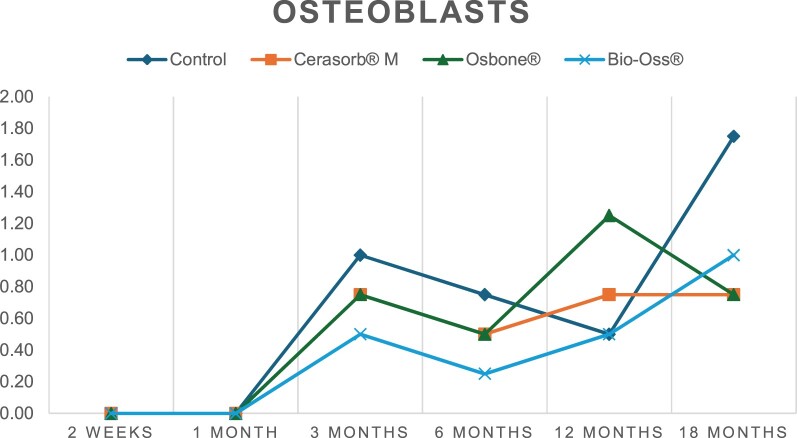
(B)	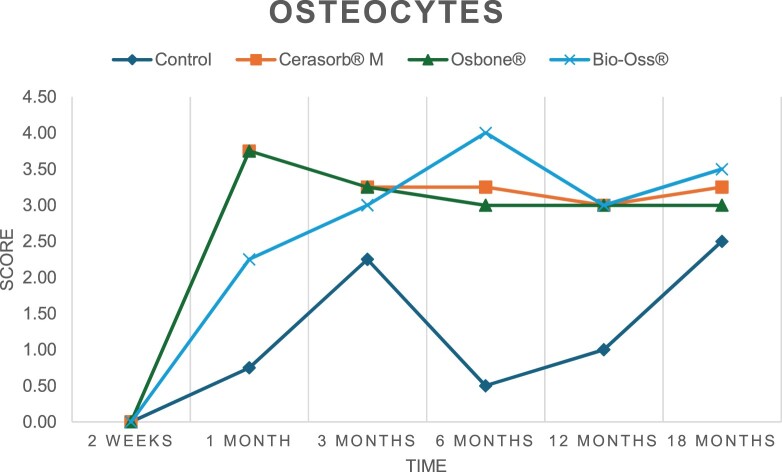
(C)	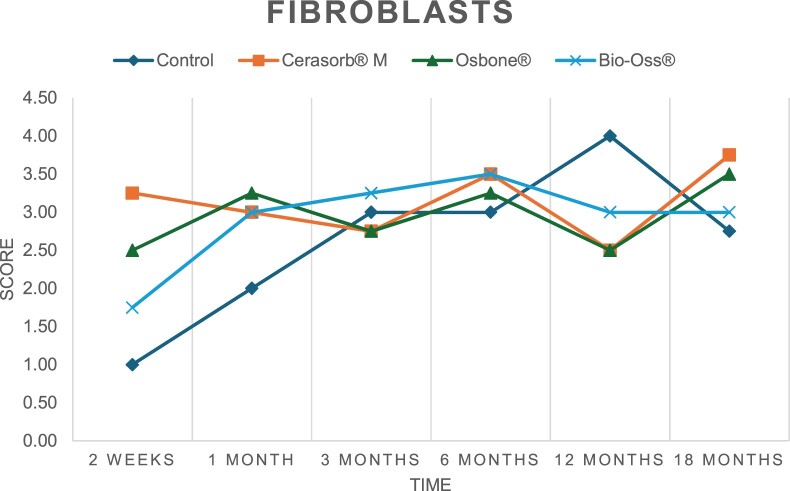
(D)	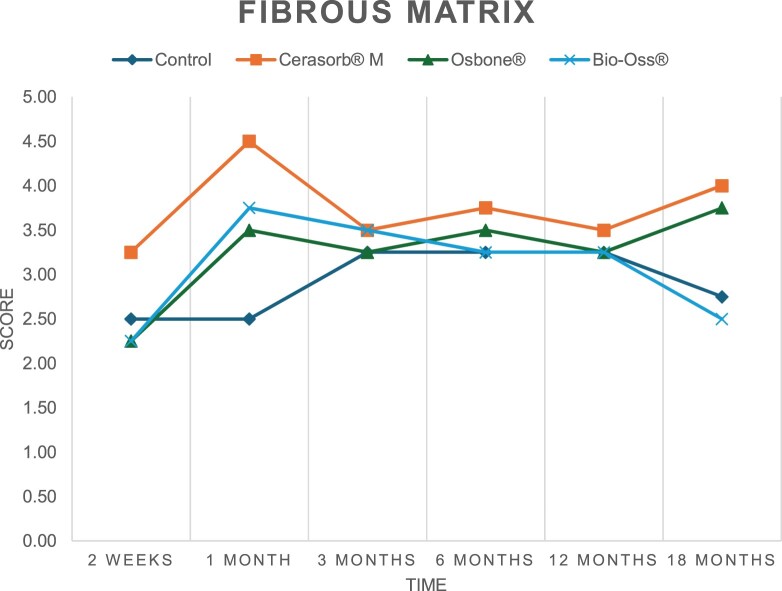
(E)	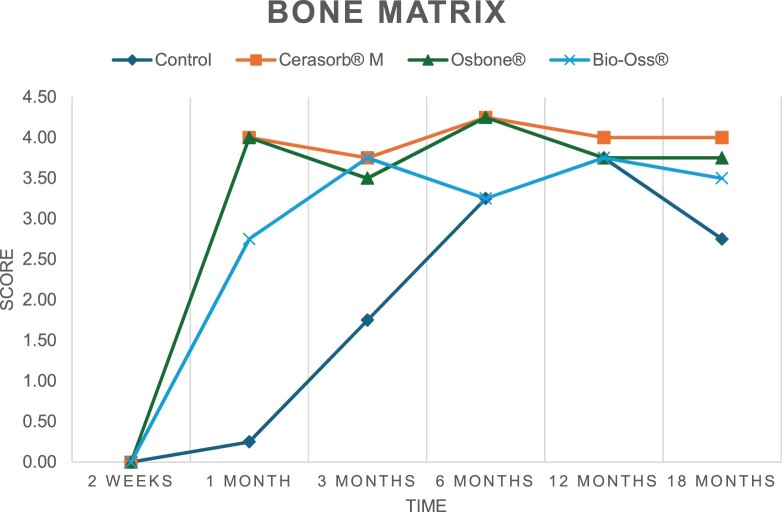
(F)	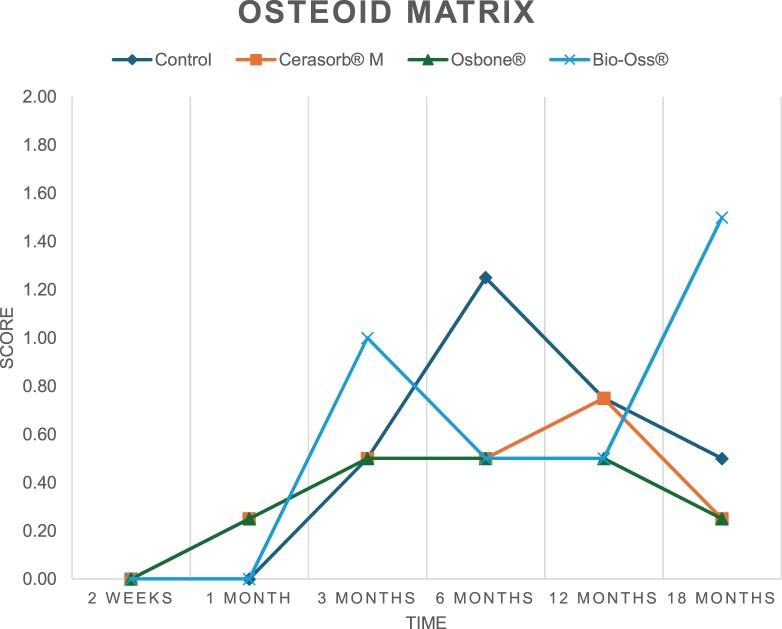

a An average score of 3.5–5 was evaluated as strong expression of a respective marker in a given cellular or matrix component, whereas an average score of (2.3–3.4), (1–2.2) and (0.1–0.9) was assessed as moderate, mild and minimal expression. All values are mean of six measurements.

**Table 3.** Line graphs depicting the mean values of the scores of alkaline phosphatase in the various cell ((A) osteoblasts, (B) osteocytes, (C) fibroblastic cells) and matrix components ((D) fibrous matrix, (E) bone matrix, (F) osteoid) at the various time points^a^

	Figure
(A)	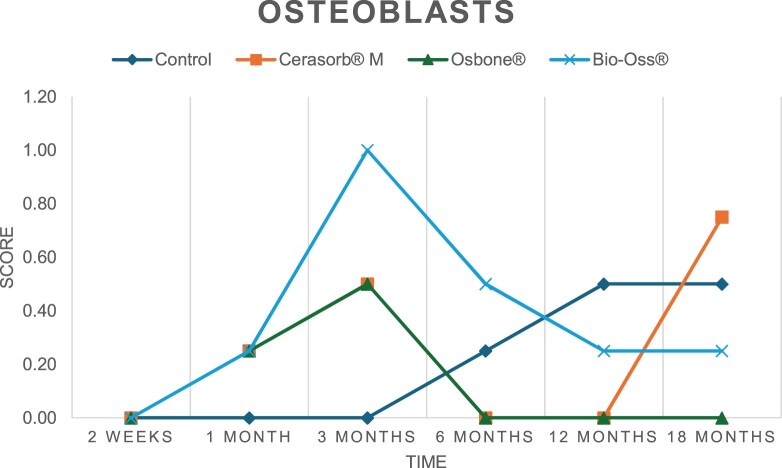
(B)	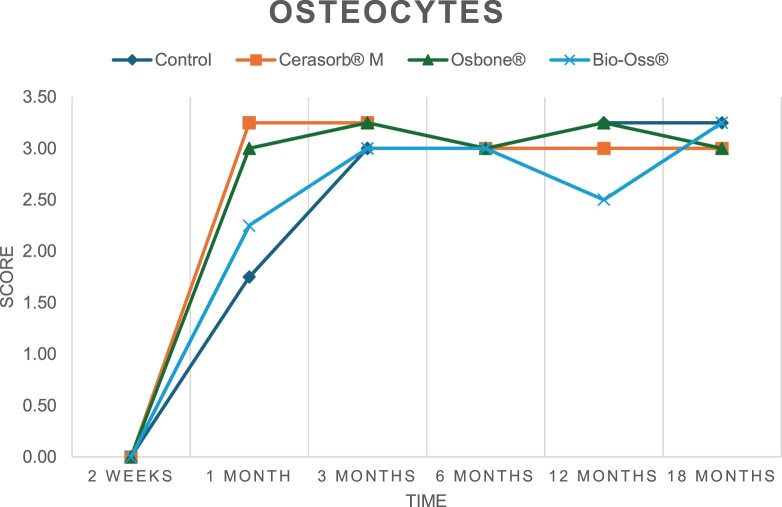
(C)	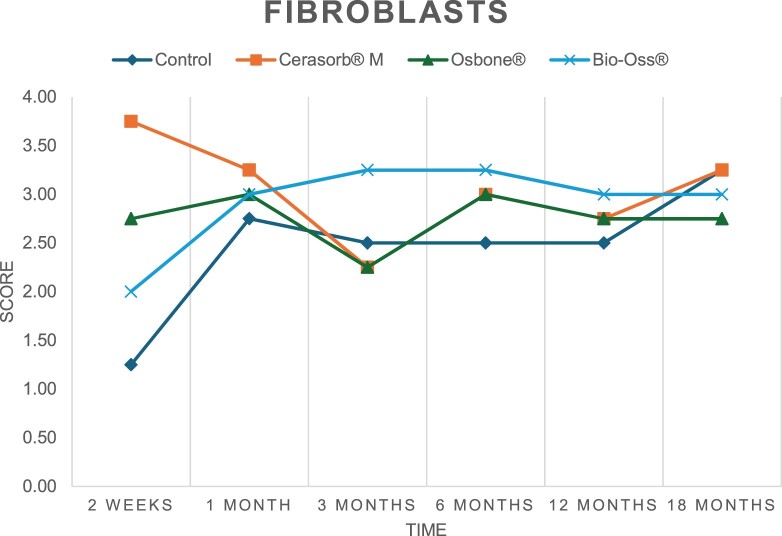
(D)	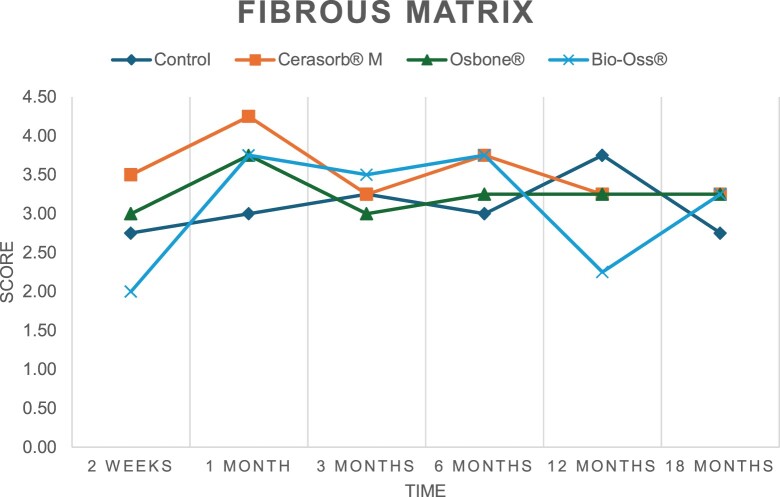
(E)	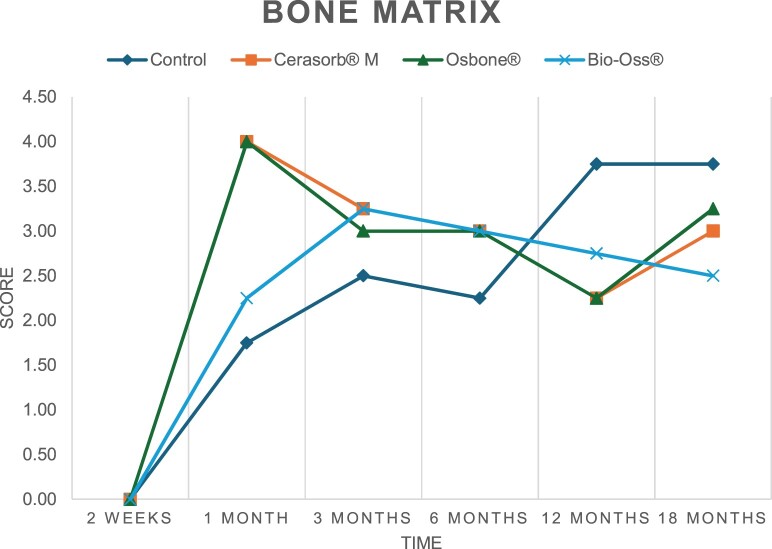
(F)	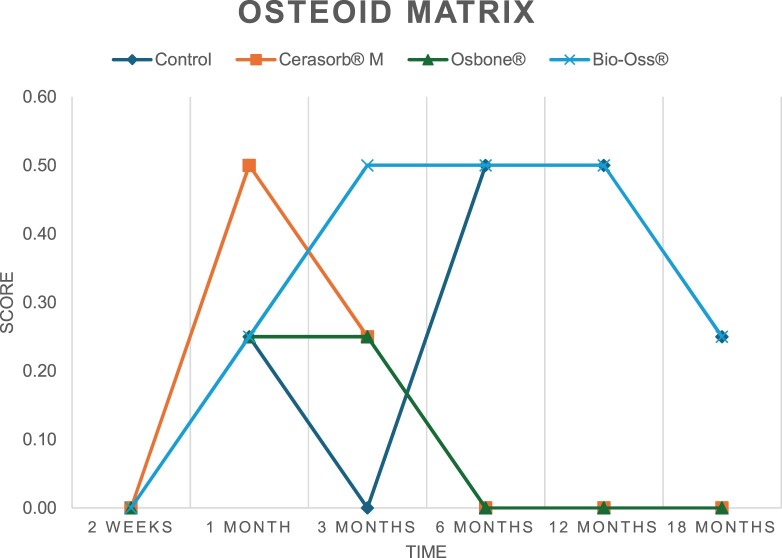

a An average score of 3.5–5 was evaluated as strong expression of a respective marker in a given cellular or matrix component, whereas an average score of (2.3–3.4), (1–2.2) and (0.1–0.9) was assessed as moderate, mild and minimal expression. All values are mean of six measurements.

**Table 4.** Line graphs depicting the mean values of the scores of type I collagen in the various cell ((A) osteoblasts, (B) osteocytes, (C) fibroblastic cells) and matrix components ((D) fibrous matrix, (E) bone matrix, (F) osteoid) at the various time points^a^

	Figure
(A)	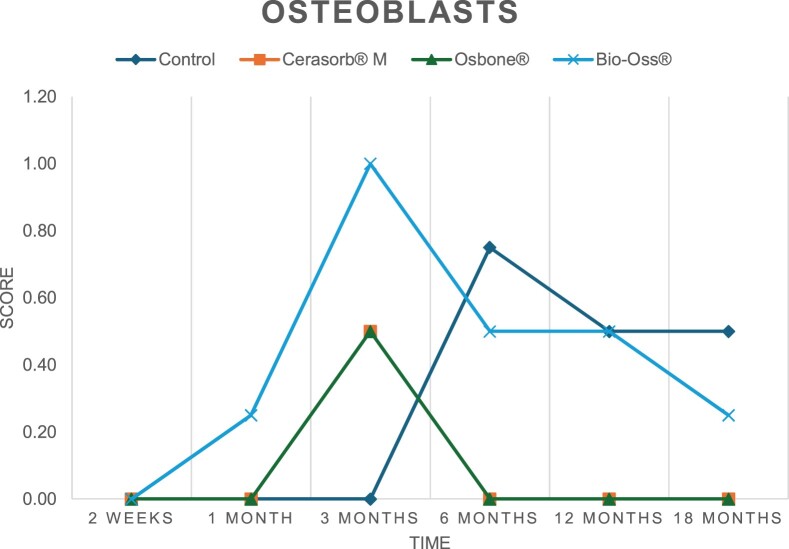
(B)	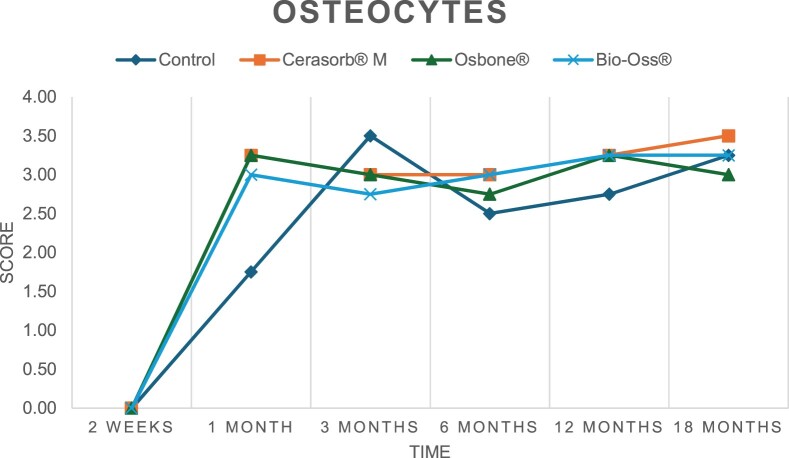
(C)	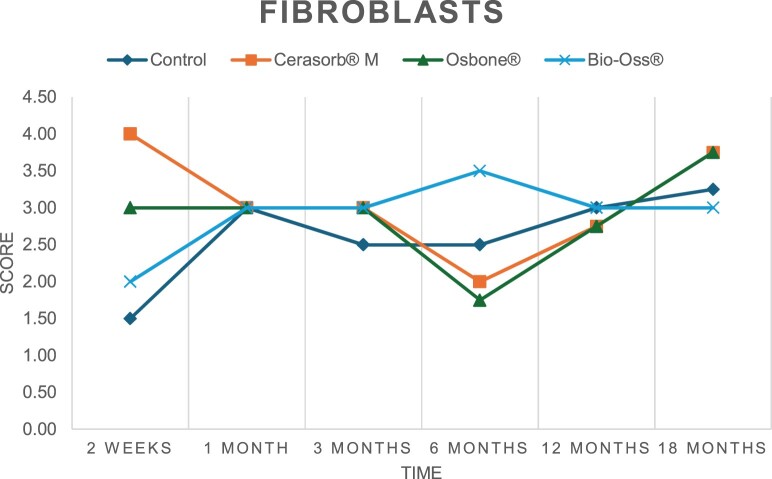
(D)	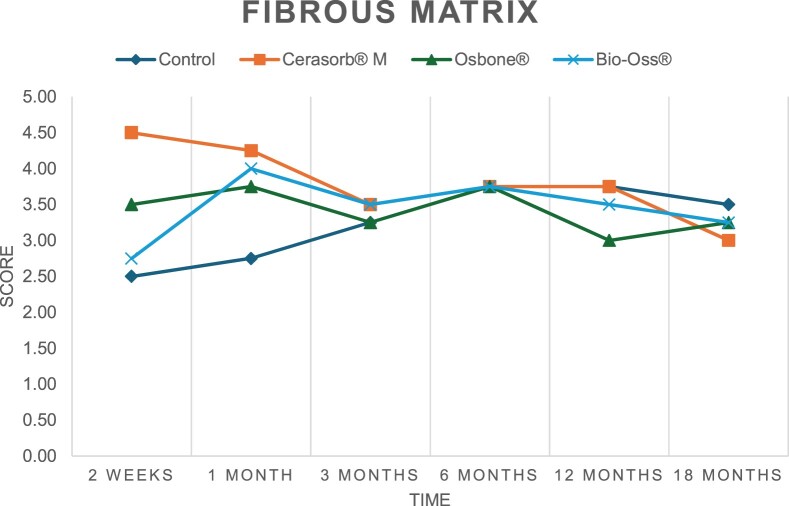
(E)	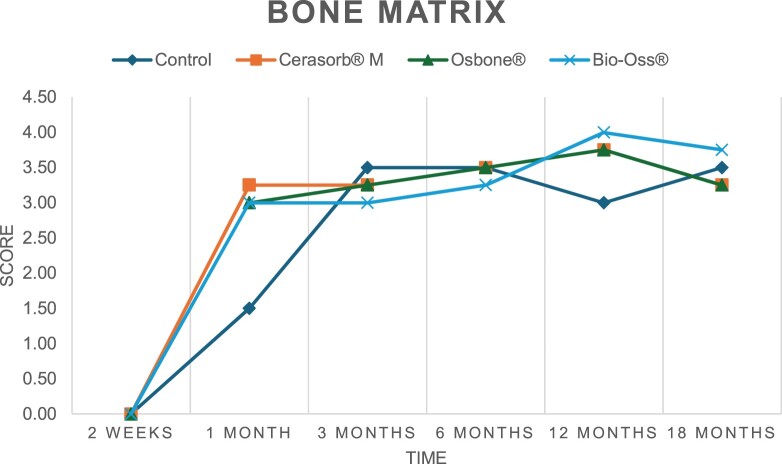
(F)	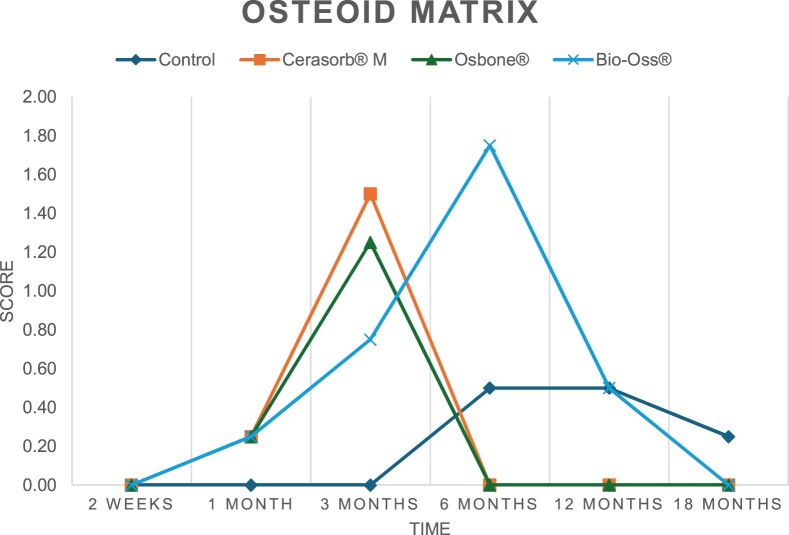

a An average score of 3.5–5 was evaluated as strong expression of a respective marker in a given cellular or matrix component, whereas an average score of (2.3–3.4), (1–2.2) and (0.1–0.9) was assessed as moderate, mild and minimal expression. All values are mean of six measurements.

**Table 5.** Line graphs depicting the mean values of the scores of bone sialoprotein in the various cell ((A) osteoblasts, (B) osteocytes, (C) fibroblastic cells) and matrix components ((D) fibrous matrix, (E) bone matrix and (F) osteoid) at the various time points^a^

	Figure
(A)	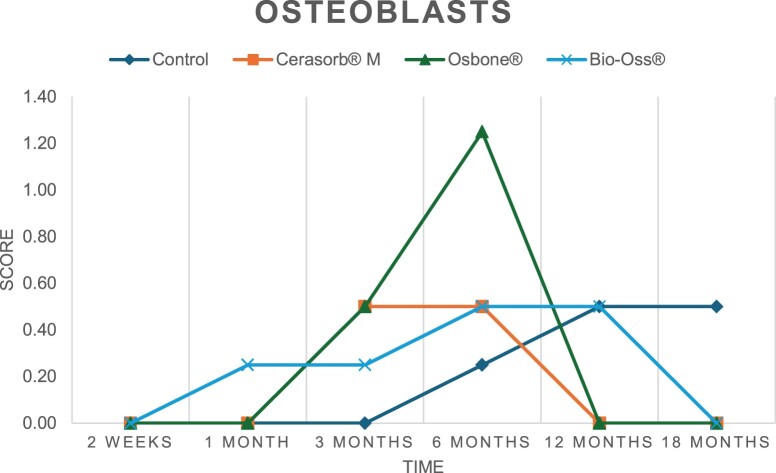
(B)	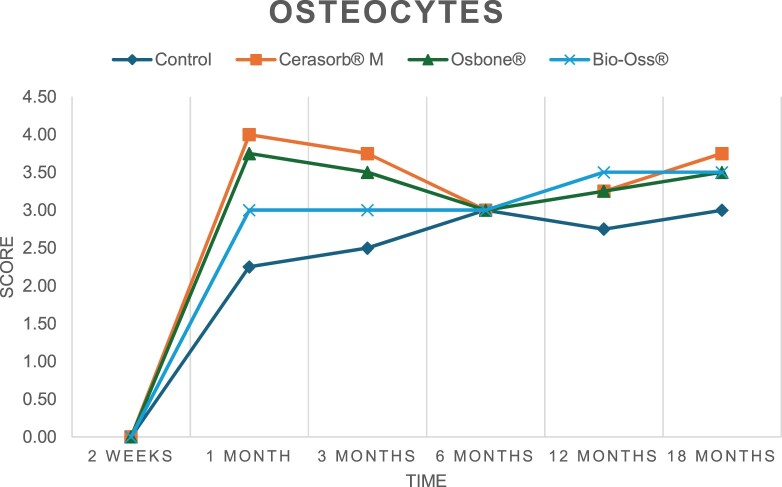
(C)	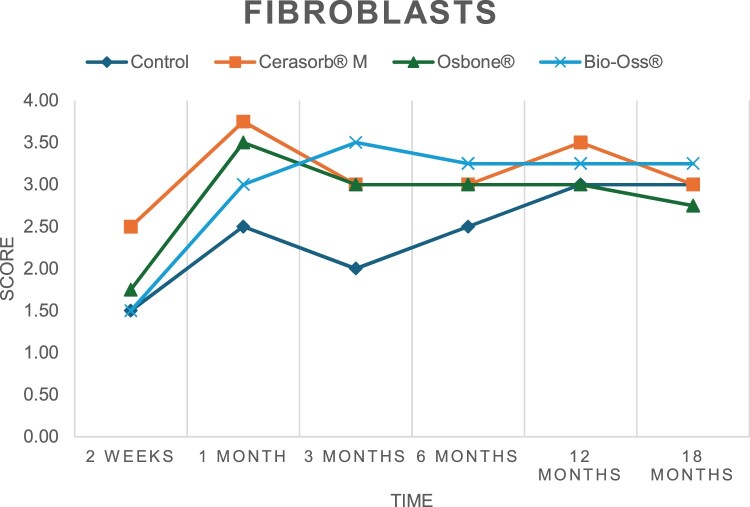
(D)	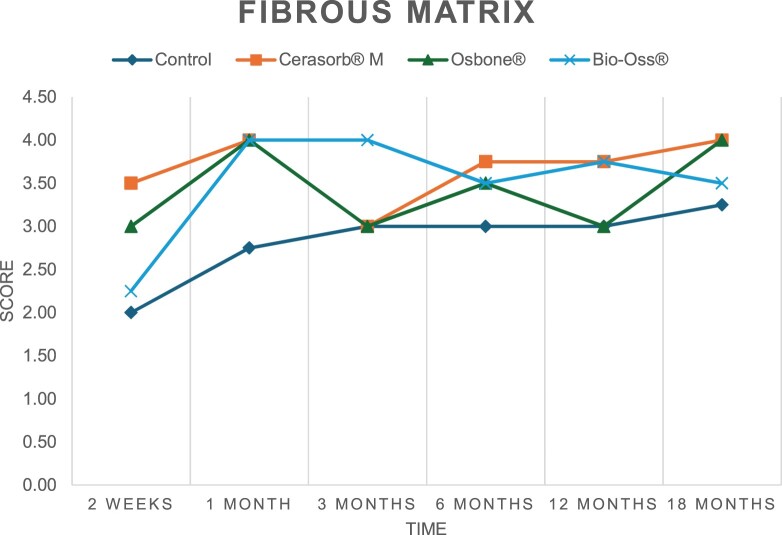
(E)	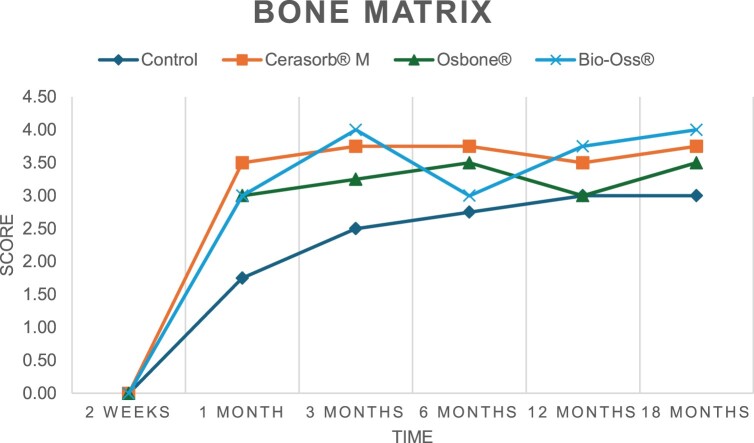
(F)	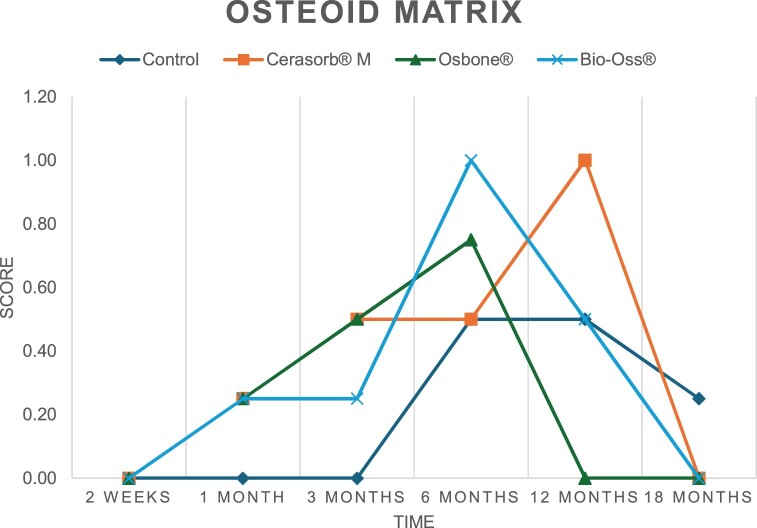

a An average score of 3.5–5 was evaluated as strong expression of a respective marker in a given cellular or matrix component, whereas an average score of (2.3–3.4), (1–2.2) and (0.1–0.9) was assessed as moderate, mild and minimal expression. All values are mean of six measurements.

**Table 6.** Representative histomicrographs of defects augmented with Osbone^®^ at different time points

Caption	Figure
At 1 month of implantation, woven bone formation is visible at the surface of the Osbone particles (green arrows). In addition, osteoblasts with moderate osteocalcin expression are present at the particle surface (white arrows). This is indicative that bone formation and matrix mineralization is actively progressing in these areas and that Osbone particles have a stimulatory effect on osteoblast differentiation, matrix mineralization and bone formation, which underscores the material’s excellent osteoconductive properties.	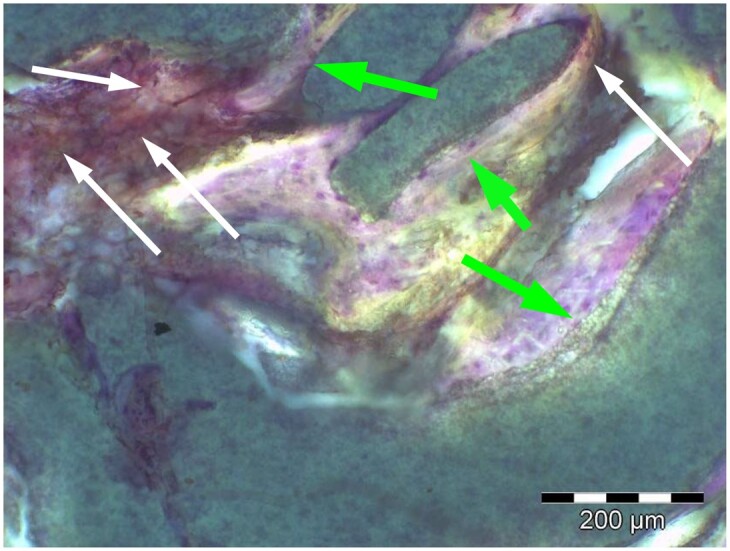
After 3 months, excellent bony regeneration after augmentation with Osbone^®^ particles becomes visible, which displays excellent bone–particle contact without any intervening soft tissue, i.e. excellent bone-bonding behaviour. Newly formed bone—green arrows, Osbone^®^ particles—orange arrows.	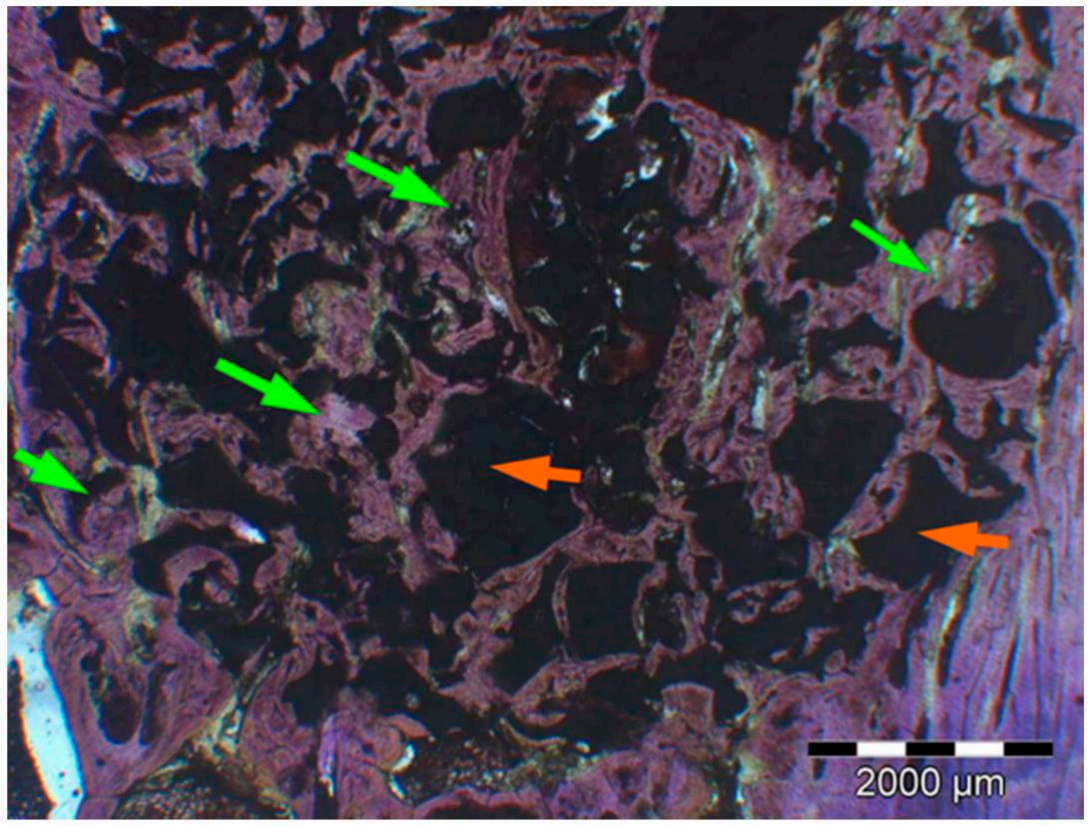
Osbone^®^ particles 3 months after implantation displaying excellent bone–particle contact (green arrows), i.e. bone-bonding behaviour. In addition, new bone formation is visible within the particles (white arrow).	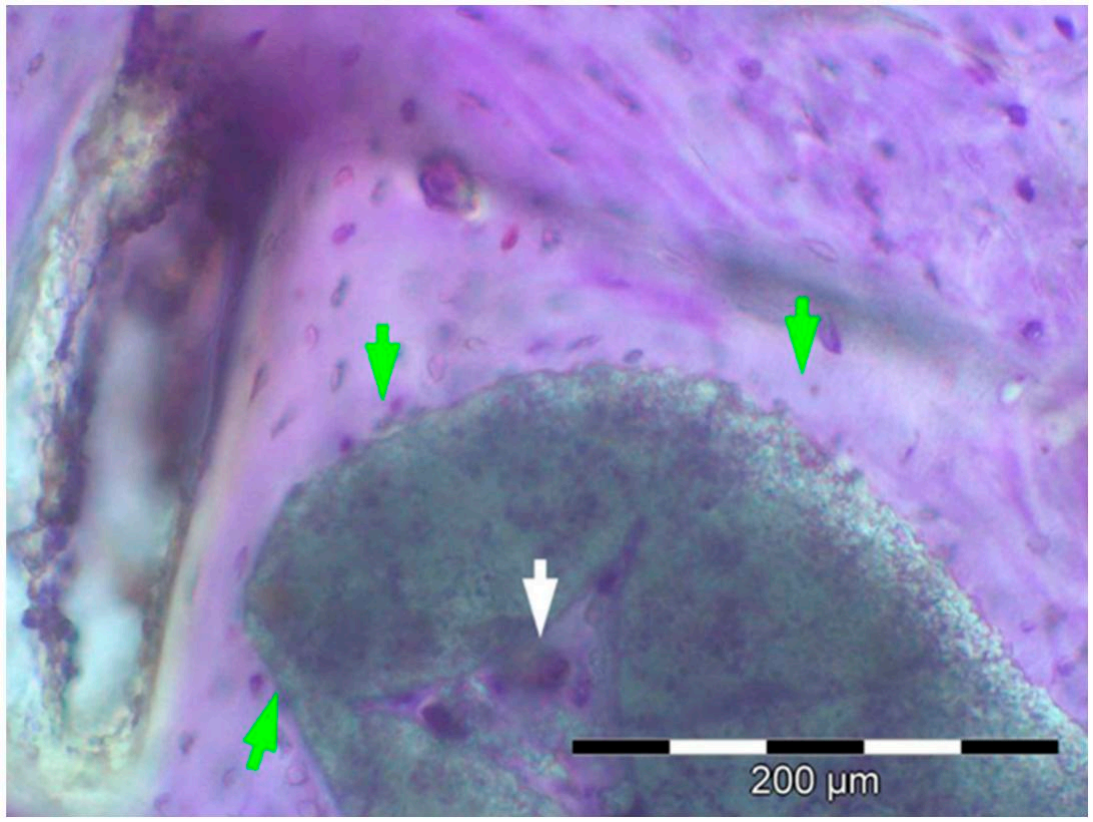
Osbone particle at 3 months displaying excellent bone-particle contact (green arrows), i.e. bone-bonding behaviour. In addition, active remodelling, and an osteoclast (pink arrow) are visible at the particle surface, this indicative that beginning resorption of the particles due to osteoclast activity and active bone remodelling with replacement by new bone is taking place in this area.	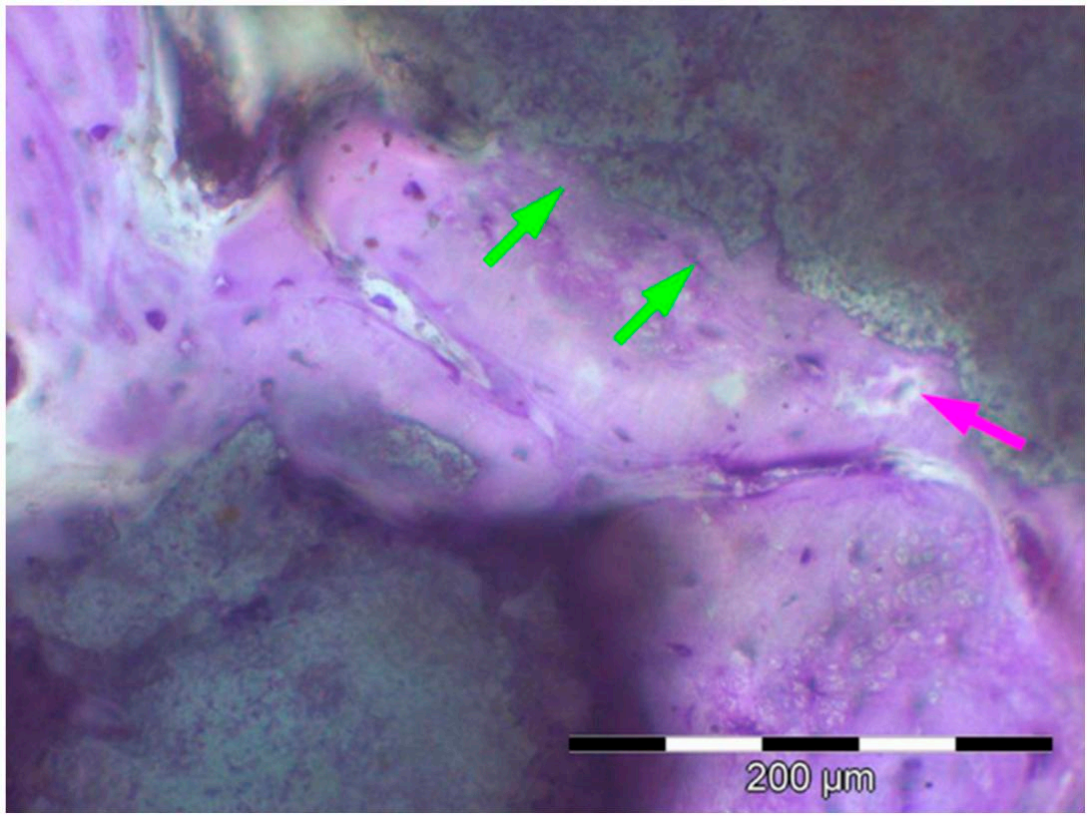
Osbone^®^ particles after 3 months of implantation in the centre of the critical-size defect displaying excellent bone-particle contact (green arrows), i.e. bone-bonding behaviour. In addition, moderate osteocalcin expression is visible in the mineralized bone matrix (yellow arrows) at the surface of the particles as well as strong osteocalcin expression in the adjacent bone marrow space (black arrow). This is indicative that matrix mineralization and bone formation and remodelling is actively progressing in these areas. It also underscores the excellent osteoconductivity and biocompatibility of the Osbone particles and their stimulatory effect on bone formation.	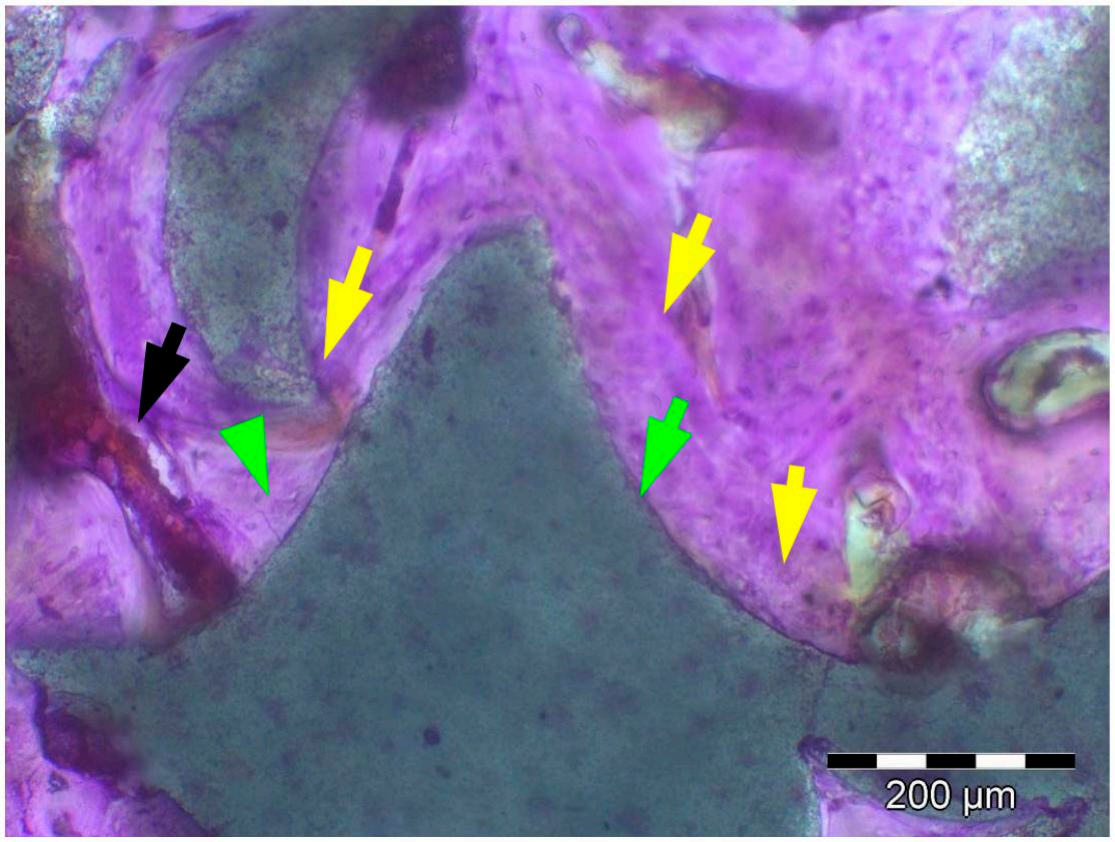
Osbone^®^ particles after 6 months of implantation displaying excellent bone–particle contact (green arrows), i.e. bone-bonding behaviour. In addition, Osbone^®^ particles show increasing degradation (orange arrows). Furthermore, strong osteocalcin expression is visible in the mineralized bone matrix (white arrows). This is indicative of actively progressing bone remodelling and matrix mineralization in these areas. It also underscores the excellent osteoconductivity and biocompatibility of the Osbone^®^ particles.	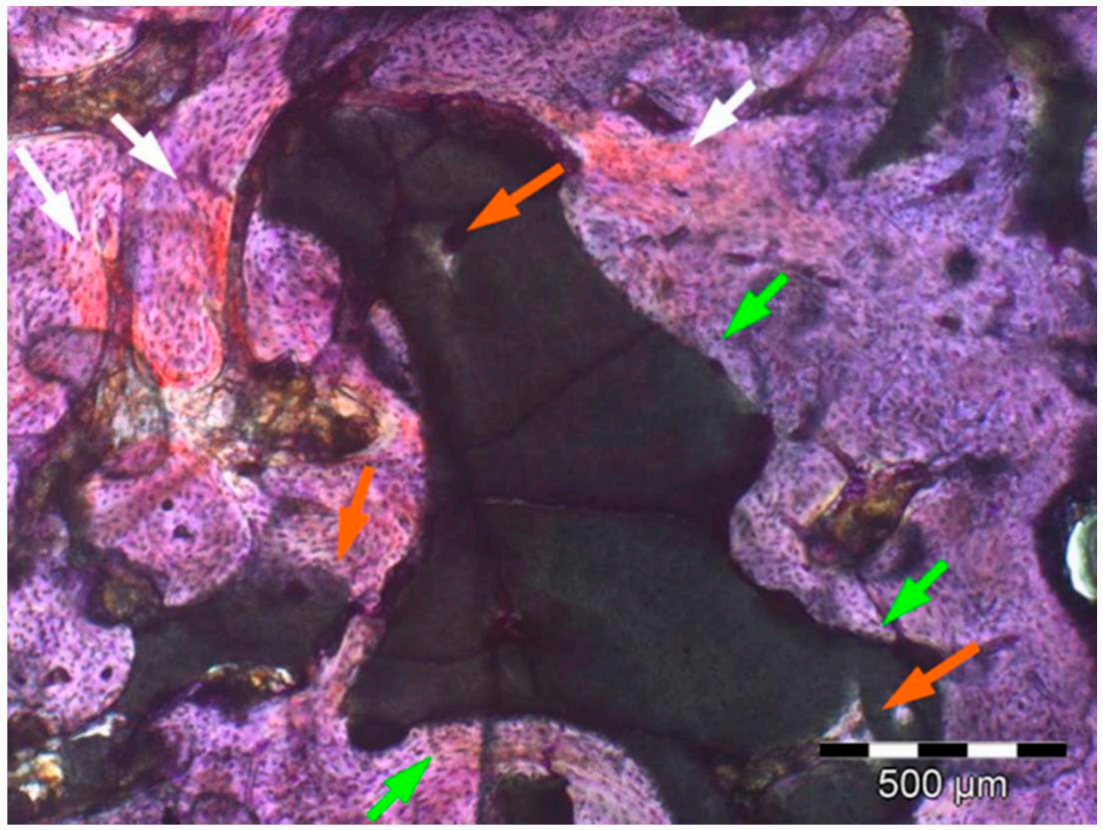
Osbone^®^ particle after 6 months of implantation displaying great bone–particle contact (green arrows), i.e. bone-bonding behaviour. In addition, strong osteocalcin expression is visible in osteoblasts (yellow arrows), which have migrated into the porosity of the Osbone^®^ particles, which show increasing degradation. Furthermore, strong staining for osteocalcin is present in the mineralized bone matrix (white arrows). This is indicative of actively progressing bone remodelling and matrix mineralization in these areas. It also underscores the great osteoconductivity and biocompatibility of the Osbone^®^ particles as well as their stimulatory effect on bone formation and good bioactive properties.	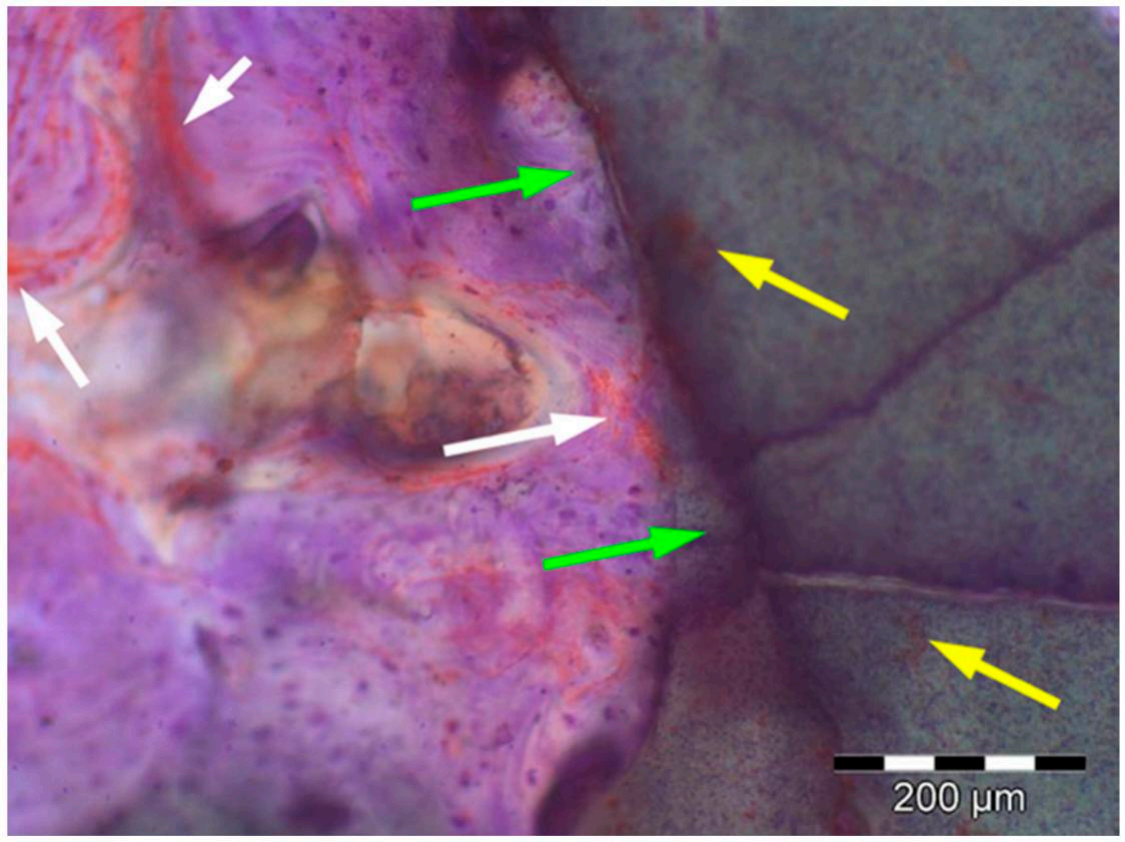
Osbone^®^ particle at 12 months displaying excellent osseous integration and bone–particle contact (green arrows), i.e. bone-bonding behaviour. In addition, increasing biodegradation is visible and extensive bone remodelling with the formation of new osteons (yellow arrows).	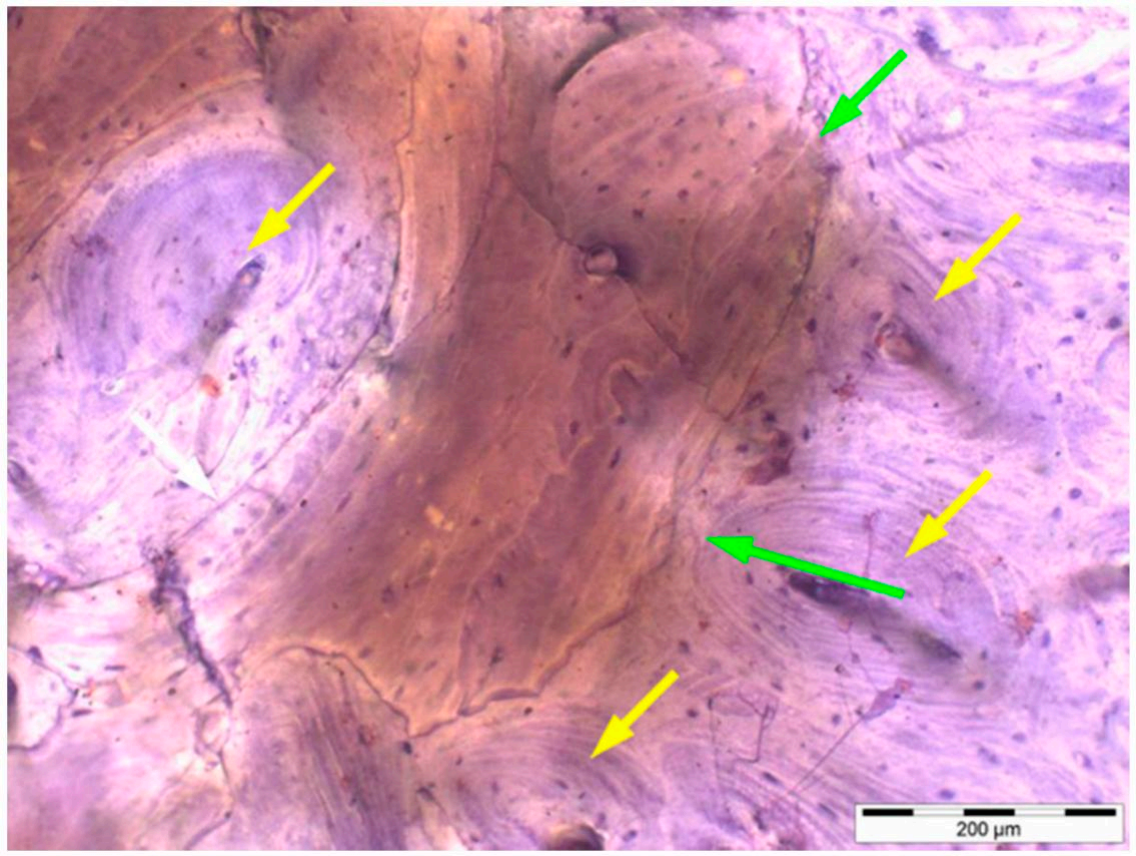
After 18 months, Osbone^®^ particles showing great bony regeneration. Osbone^®^ particles display good osseous integration and bone–particle contact (green arrows), i.e. bone-bonding behaviour.	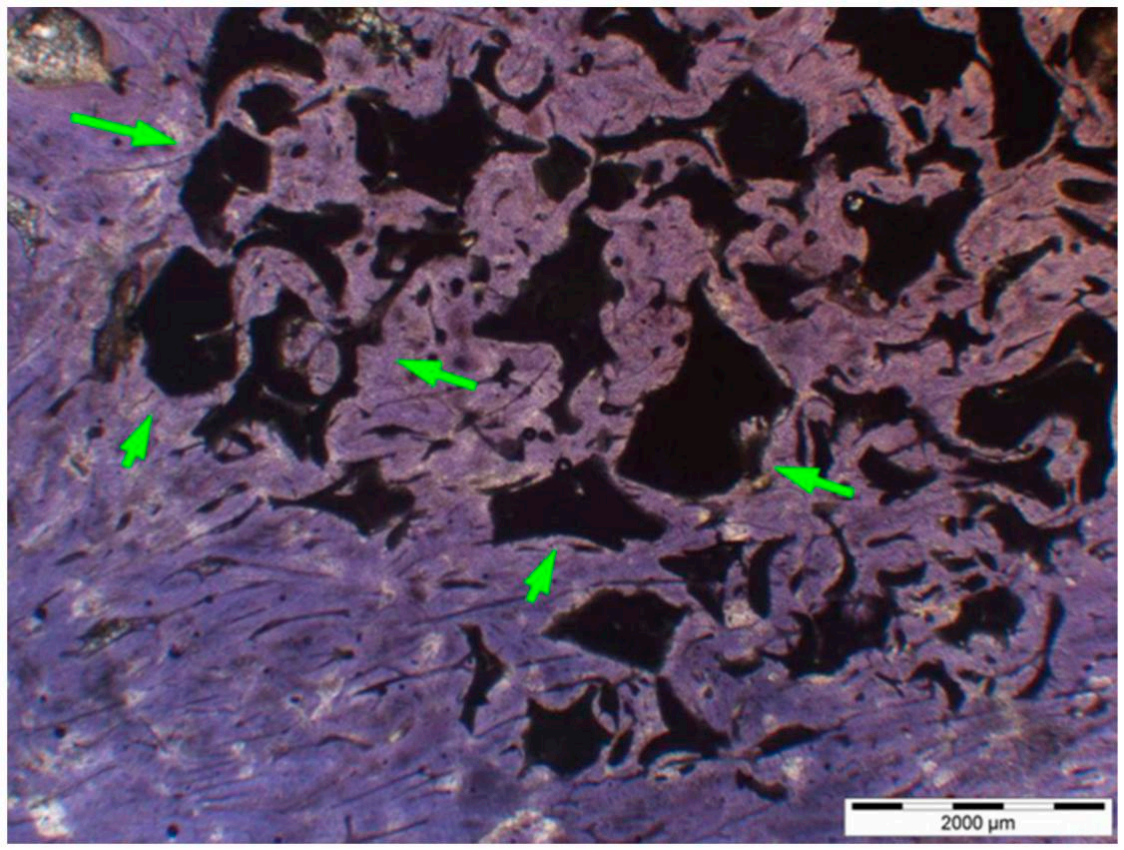
Osbone^®^ particles at 18 months displaying excellent osseous integration and bone–particle contact (green arrows), i.e. bone-bonding behaviour. In addition, increasing biodegradation is visible (white arrows) along with moderate to strong expression of bone sialoprotein (yellow arrows) of the mineralized bone matrix, indicating areas of bone remodelling.	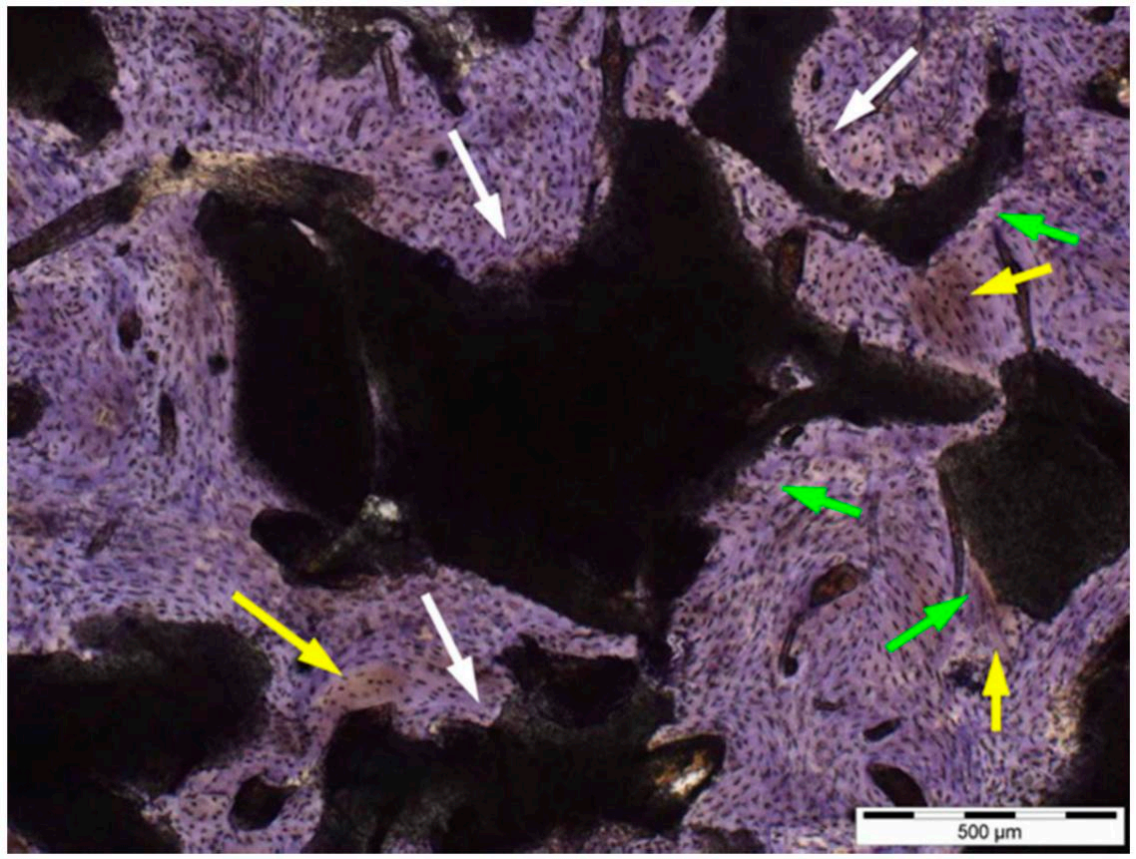

**Table 7.** Representative histomicrographs of defects augmented with Bio-Oss^® ^at different time points

Caption	Figure
Bio-Oss^®^ particles at 1 month, with beginning bone formation at their surface in various areas (green arrows).	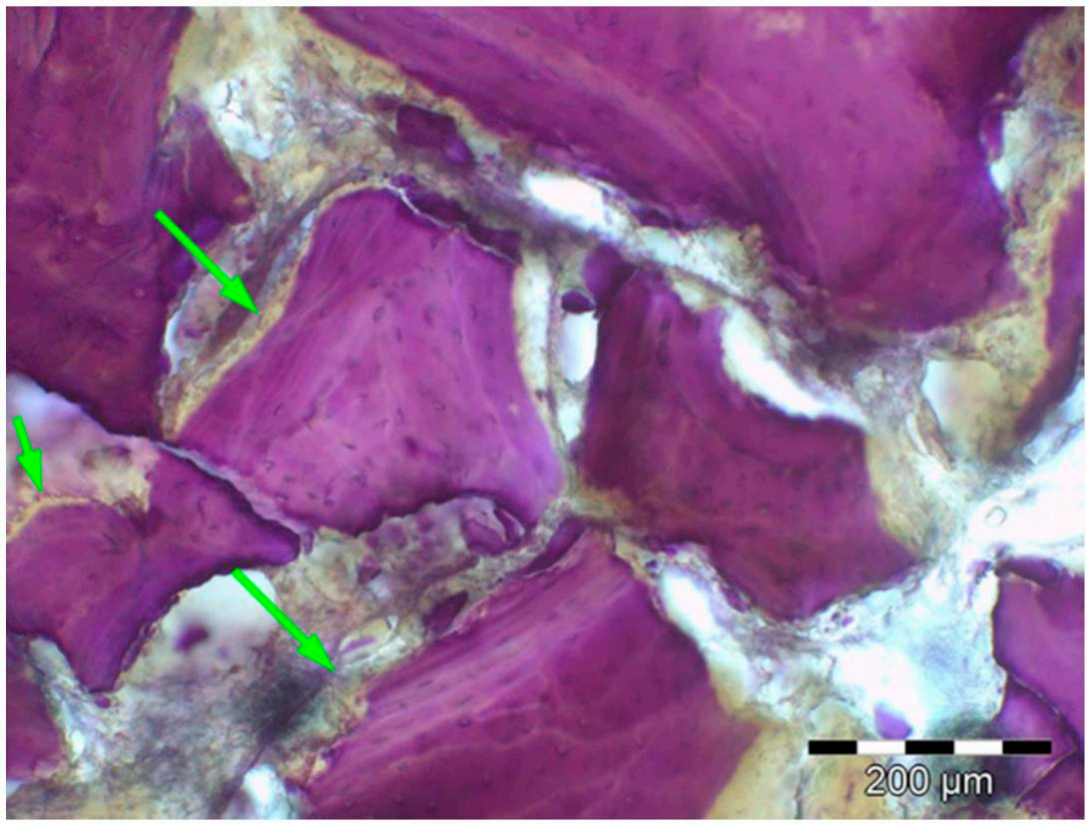
After 3 months, great bony regeneration of the defect has occurred with Bio-Oss^®^ particles displaying high bone–particle contact (green arrows), i.e. excellent bone-bonding behaviour.	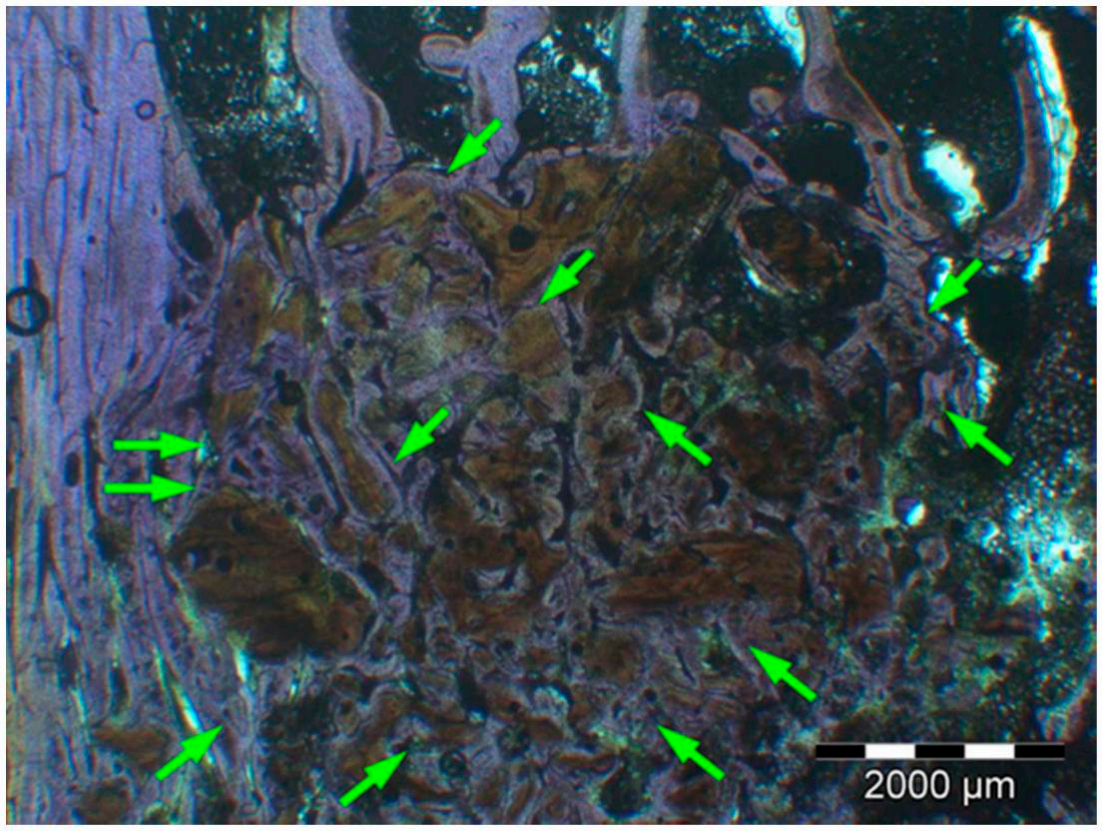
After 3 months, the particles display high bone–particle contact (green arrows), i.e. excellent bone-bonding behaviour. In addition, strong osteocalcin expression is present in the cells (fibroblasts and osteoblasts) and tissue of the adjacent bone marrow spaces (black arrows) in contact with the Bio-Oss^®^ particles. This indicates that bone formation, matrix mineralization and bone remodelling are actively progressing at 3 months and underscores the excellent biocompatibility and osteoconductivity of the Bio-Oss^®^ particles.	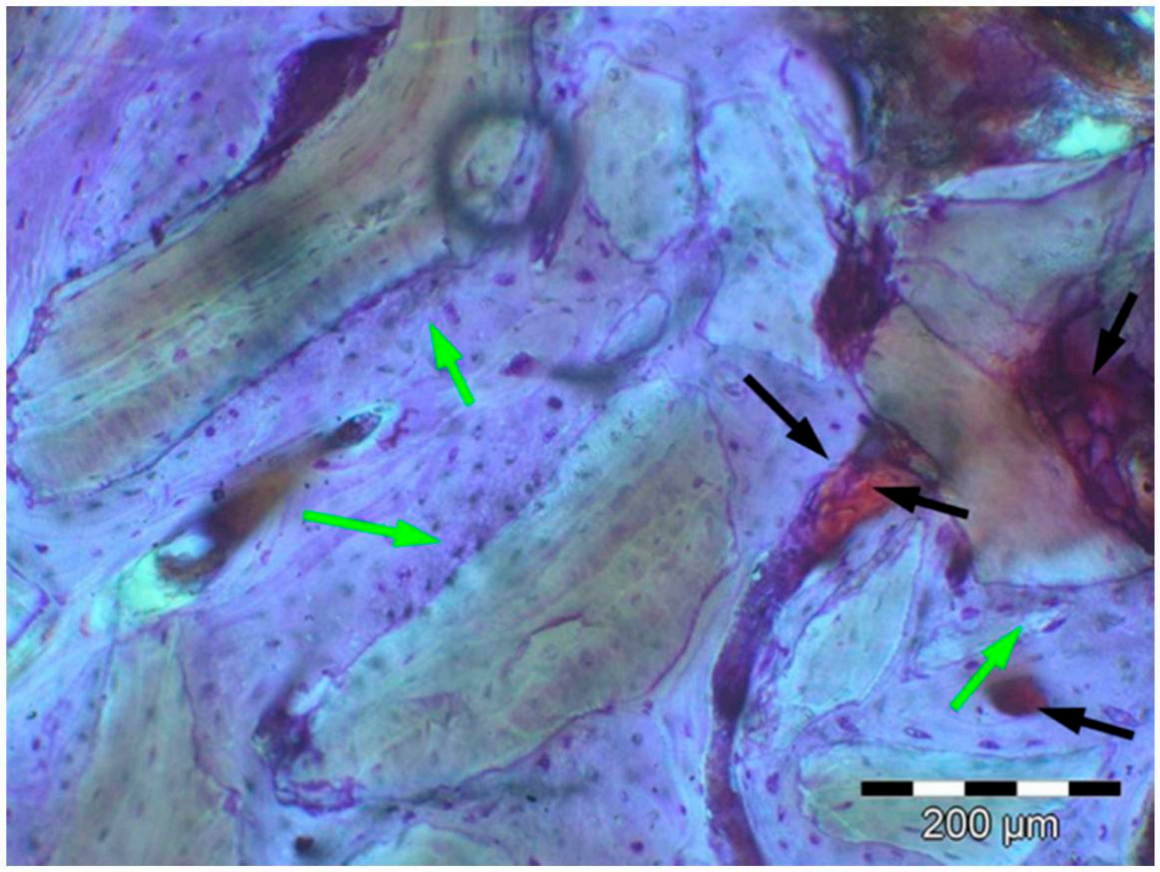
After 6 months, the Bio-Oss^®^ particles display high bone–particle contact (green arrows), i.e. excellent bone-bonding behaviour. In addition, strong bone sialoprotein expression is present in osteoblasts (orange arrow) as well as moderate bone sialoprotein expression of the mineralized bone matrix (yellow arrows). This indicates that at 6 months, bone formation and remodelling, including matrix mineralization, are actively progressing in these areas.	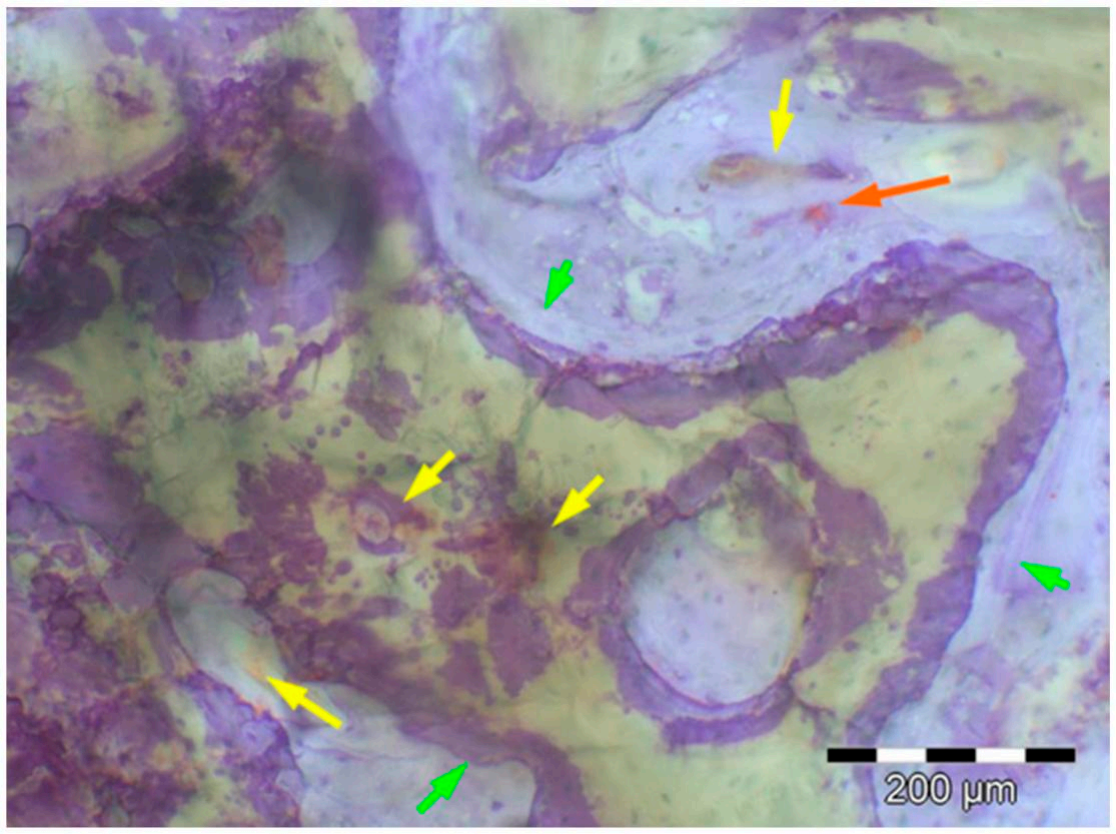
12 months after augmentation with Bio-Oss^®^ particles, which display excellent osseous integration and bone–particle contact (green arrows), i.e. bone-bonding behaviour. Moreover, excellent bony regeneration and bone remodelling of the defect have occurred.	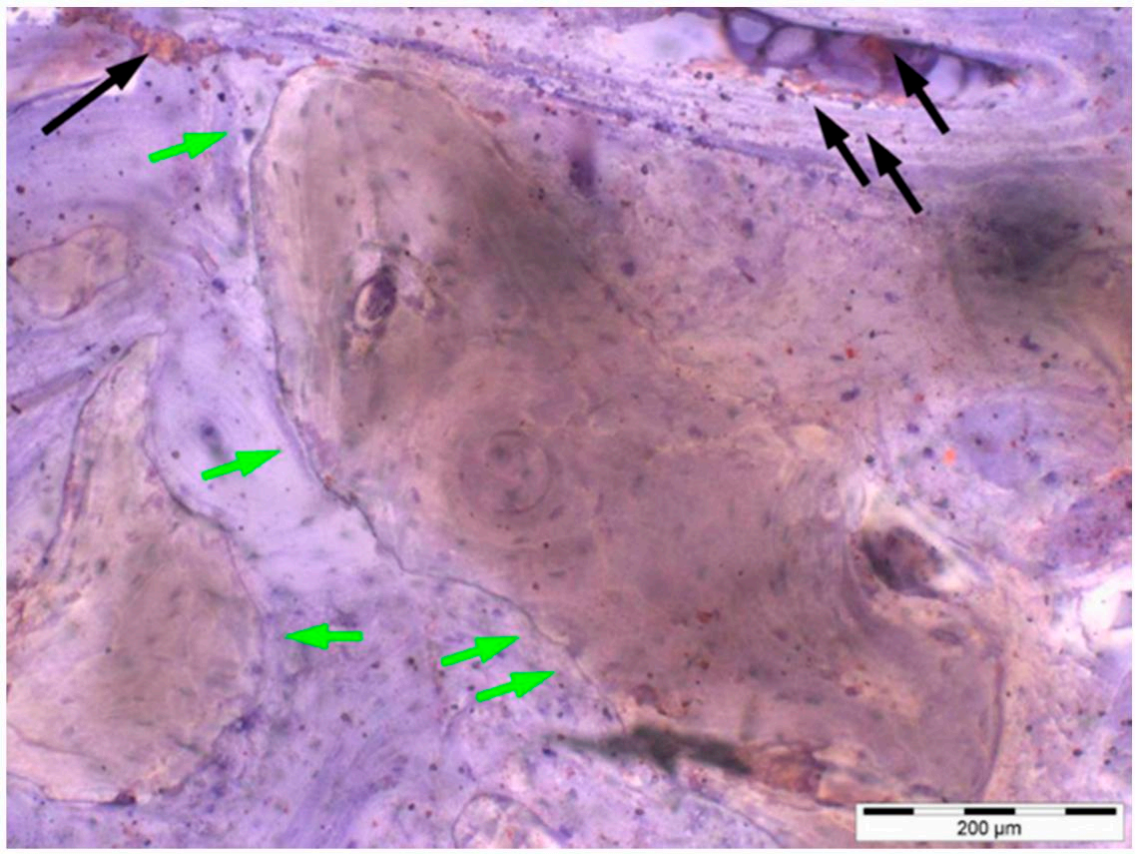
18 months after augmentation with Bio-Oss^®^ particles, which display excellent osseous integration and bone–particle contact (green arrows), i.e. bone-bonding behaviour. Moreover, excellent bony regeneration and bone remodelling of the defect have occurred.	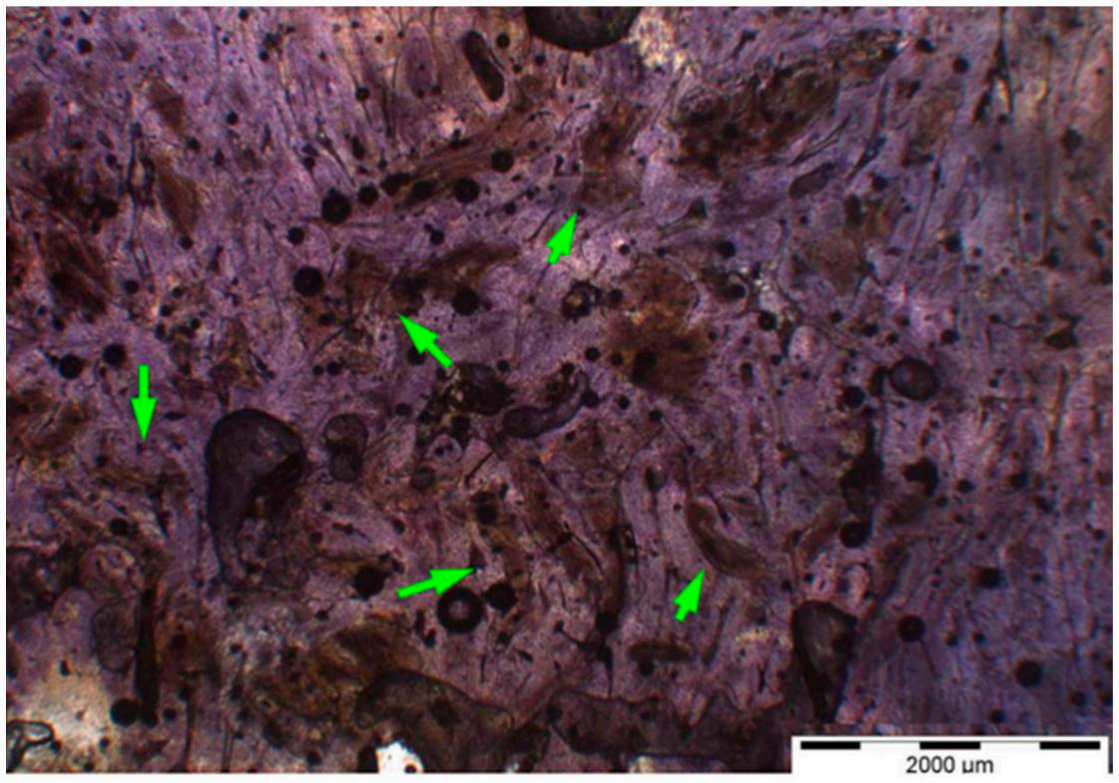
After 18 months of implantation, the particles display high bone–particle contact (green arrows), i.e. excellent bone-bonding behaviour. In addition, areas of partial particle degradation and replacement by new bone are visible (yellow arrows).	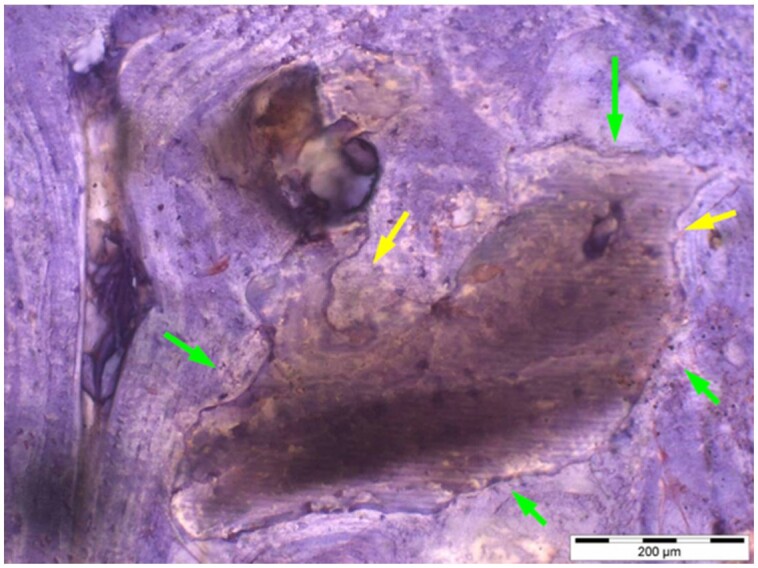
Empty control defect 18 months after surgery. The defect is mainly filled by scar tissue (white arrow). Very limited bone formation (yellow arrow) has occurred at the defect margins over the 18-month healing period.	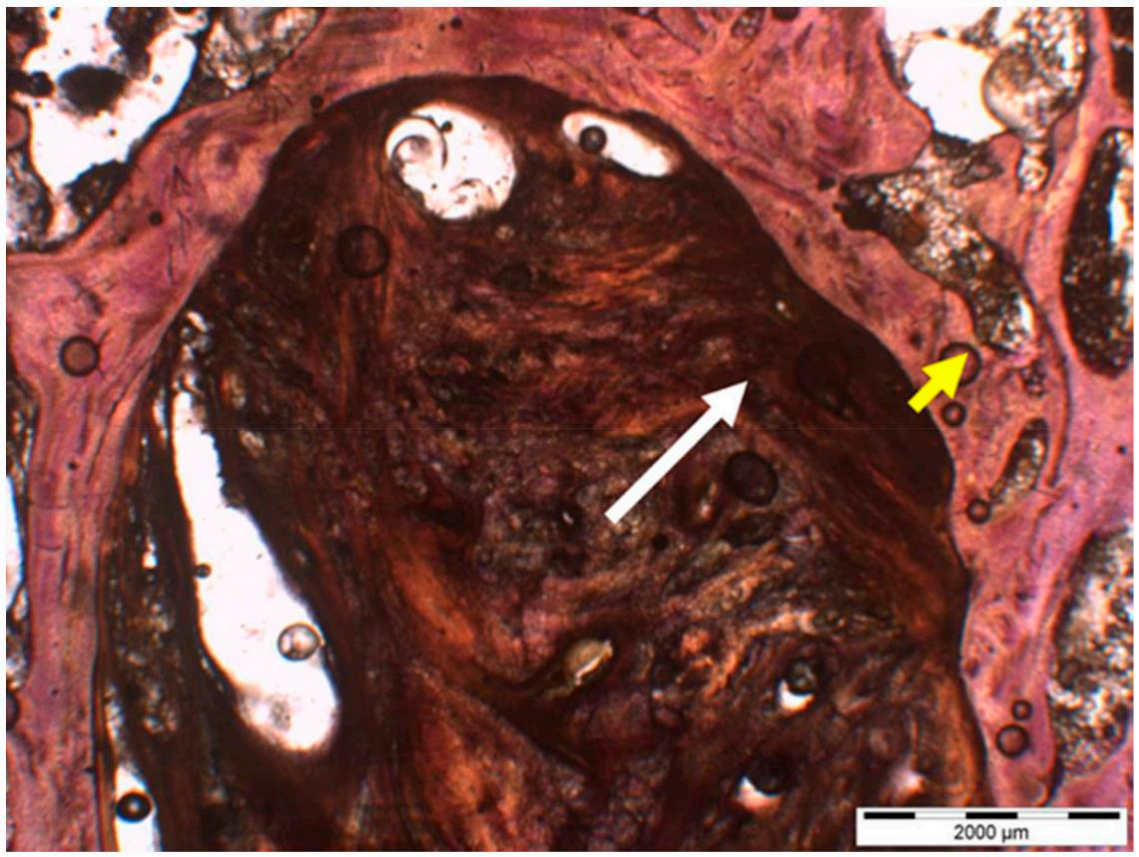

**Table 8.** Representative histomicrographs of defects augmented with Cerasorb^®^ M at different time points

Caption	Figure
After 3 months of implantation, the new bone formation has occurred within the degrading Cerasorb^®^ M particles (green arrows), which display great bone–particle contact, i.e. bone-bonding behaviour, without any intervening soft tissue (orange arrows).	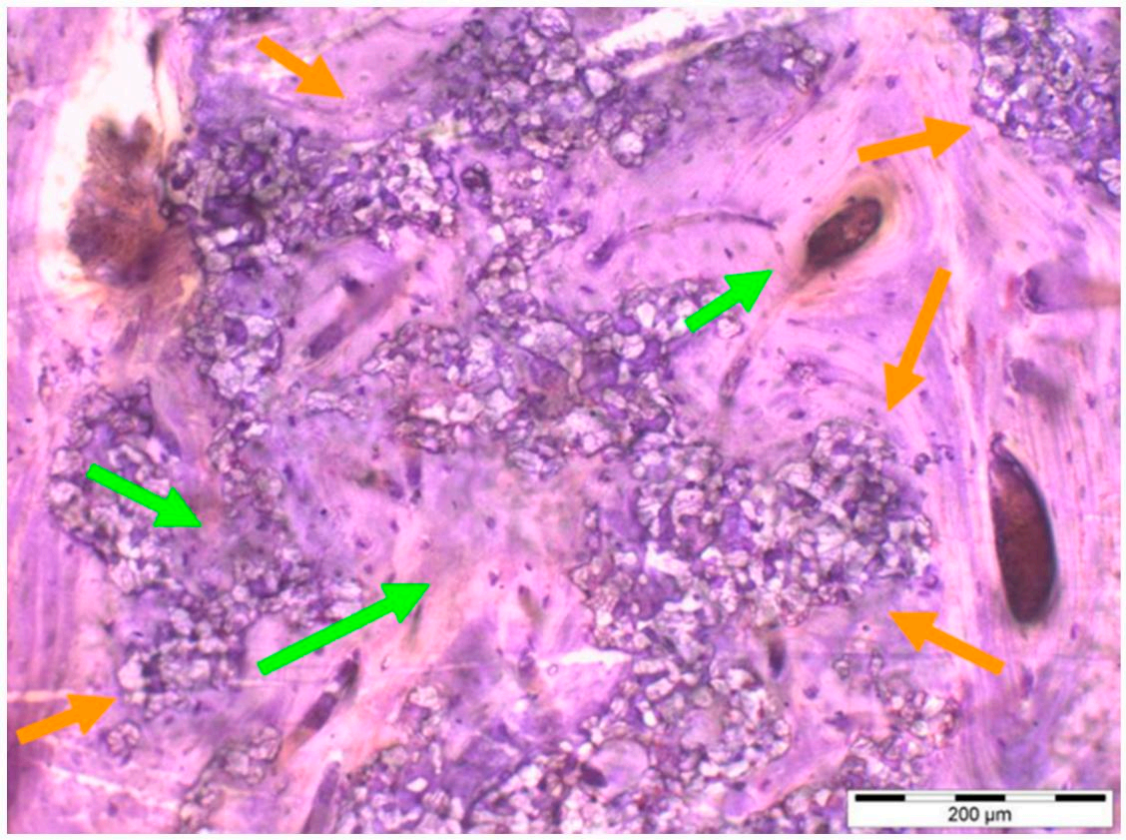
After 6 months, residual partially degraded Cerasorb^®^ M particles display good bony integration and bone-bonding behaviour (green arrows). In addition, areas of bone remodelling with positive expression of the osteogenic marker osteocalcin in the mineralized bone matrix are visible (orange arrows), which indicates new bone formation and matrix mineralization taking place in these areas.	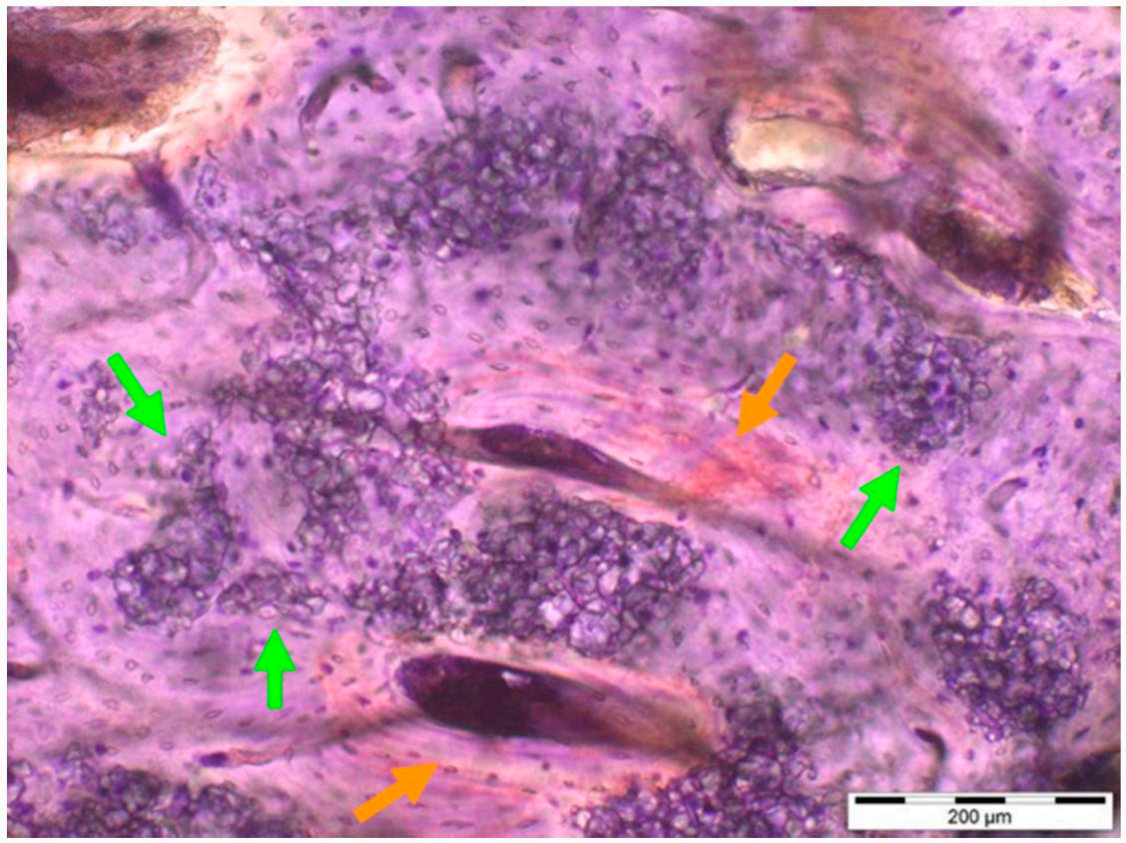
At 12 months, remodelling of the defect has further progressed with increasing restoration of the original cancellous bone structure with bone marrow spaces (yellow arrows). At the same time, particle degradation has also progressed further with residual particle fragments, which display great bone-bonding behaviour, i.e. bone–particle contact (green arrows) is still present.	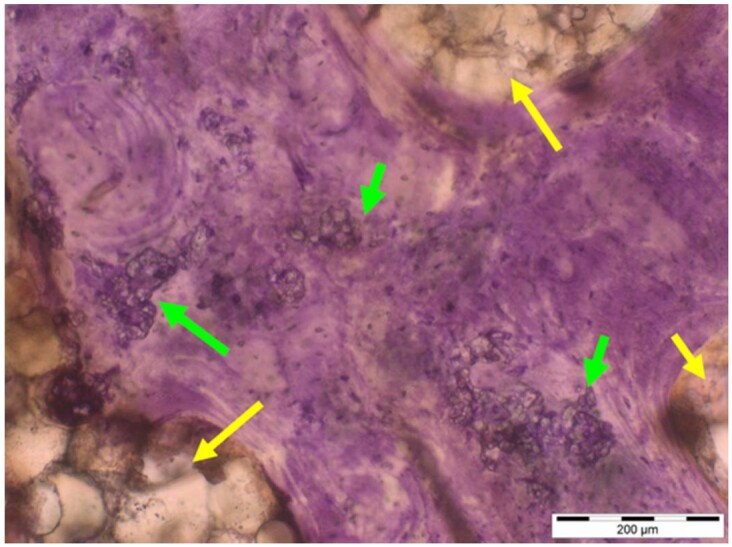
Highly degraded Cerasorb^®^ M fragments are visible 18 months after implantation, which display excellent bone–particle contact, i.e. bone-bonding behaviour, and formation of an osteon in the centre of the particle residues (yellow arrow). Positive staining for the osteogenic marker bone sialoprotein (blue arrows) is indicative of actively progressing bone remodelling and particle degradation in this area.	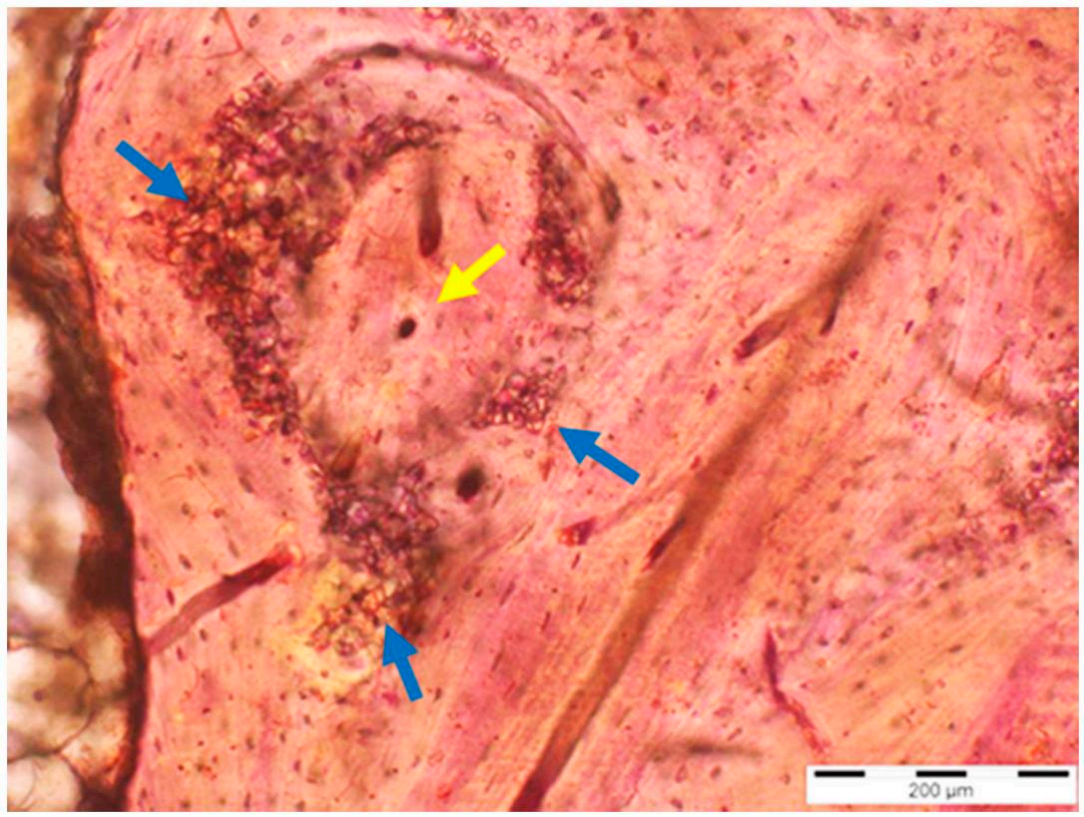

After *2 *weeks, new bone formation with no significant differences (*p *>* *0.05) was observed in all augmented defects, with β-TCP showing less newly formed bone (mean particle area fraction 0.76 ± 0.60%) than both defects augmented with HA (Bovine HA (BHA) 1.04 ± 0.21%; synthetic HA (SHA) 1.54 ± 1.44%). Both HA and β-TCP particles were mainly surrounded by the fibrous matrix of the osteogenic mesenchyme tissue, with new woven bone formation being visible at the particle surface of TCP and SHA and at the defect margins, respectively. Strong osteocalcin (OC) expression in this fibrous matrix (Table 2) suggested the onset of bone matrix mineralization in these areas for all three materials. The highest expression levels of ALP (Table 3) and type I collagen (Col I) (Table 4) were noted in defects grafted with β-TCP, whereas levels for BSP were comparable for all groups (Table 5). The bone–particle contact observed in defects augmented with β-TCP and synthetic HA was similar, with values of 0.40 ± 0.56% and 0.41 ± 0.22%, respectively, whereas no bone biomaterial contact was visible in defects augmented with the bovine-derived HA (0.0 ± 0.0%). No bone formation was recorded in empty controls.

After 1 month, the test material granules were surrounded by newly formed woven bone in all augmented defects, with high levels of OC (Table 2) being noted in the bone matrix of β-TCP and synthetic HA augmented defects, indicating active-matrix mineralization in these regions, while bovine-derived HA augmented defects showed moderate levels of OC in the bone matrix, suggesting less advanced bone formation. The expression levels of ALP (Table 3) showed a similar pattern to OC, and the expression level of BSP and Col I was similar in all three groups (Tables 4 and 5). New bone formation was significantly higher in all augmented defects when compared to the empty defects (β-TCP 41.73 ± 1.25%, SHA 36.29 ± 0.37%, BHA 17.32 ± 1.20, ED 5.50 ± 0.79%; *p *≤* *0.01), whereby the greatest bone formation (41.73 ± 1.25%) in combination with a high bone–particle contact (80.91 ± 3.72%) and degradation (residual amount of grafting material, i.e. particle area fraction 33.75 ± 3.35%) was observed in defects augmented with β-TCP. The bone–particle contact was significantly lower for bovine-derived HA compared to β-TCP and synthetic HA (*p *≤* *0.01). When comparing both HA-based materials, higher bone formation (SHA 36.29 ± 0.37 vs. BHA 17.32 ± 1.20%; *p* ≤ 0.02 ) and bone–particle contact (SHA 60.99 ± 3.45% vs BHA 16.88 ± 5.24%) was recorded for defects augmented with synthetic HA. The bovine-derived HA, however, exhibited fewer residual particles (SHA 50.11 ± 0.48 vs. BHA 38.65 ± 1.41%; *p *≤* *0.04).

After 3 months, particle degradation, bone formation and defect regeneration had progressed further in all augmented defects. The β-TCP particles facilitated excellent bone regeneration and displayed increasing biodegradation as well as bone–particle contact without any intervening soft tissue formation. As such, defects grafted with β-TCP exhibited the greatest bone formation (β-TCP 55.65 ± 2.03% vs. SHA 49.05 ± 3.84% and BHA 47.59 ± 1.97%; *p *≤* *0.03) in combination with the highest particle degradation (β-TCP 24.58 ± 2.23% vs. SHA 47.92 ± 1.18% vs. BHA 33.79 ± 1.20%; *p *≤* *0.02) among all tested materials. Moreover, all bone substitute materials showed high bone–particle contact without any statistically significant differences (β-TCP 95.63 ± 2.72% vs. SHA 95.33 ± 1.85% vs. BHA 91.86 ± 8.28%; *p* > 0.05). OC and BSP expression (Tables 2 and 5), especially in the bone matrix, was comparable within all three groups.

After 6 months, β-TCP particle degradation and bone regeneration had proceeded further with increasing bone remodelling into lamellar and cancellous bone. At the same time, high OC expression was noted in the bone matrix, which is indicative of high remodelling activity. The greatest bone formation was noted in β-TCP grafted defects (β-TCP 62.03 ± 1.58%; SHA 55.83 ± 2.59%; BHA: 53.44 ± 0.78%; *p *≤* *0.04) when compared to defects augmented with the HA-based materials. While β-TCP displayed the smallest number of residual particles (β-TCP 23.74 ± 0.40%; BHA: 30.72 ± 1.10%; SHA 38.52 ± 0.89%; *p* > 0.05), all grafting materials exhibited similarly high bone–particle contact (β-TCP 96.35 ± 3.02%; SHA 96.30 ± 2.15%; BHA 92.36 ± 5.36%; *p* > 0.05) without any statistically significant differences.

Bone formation and remodelling progressed further after 12 months, with a slightly higher amount of bone being present in β-TCP grafted defects than in defects augmented with HA-based materials (β-TCP 75.42 ± 1.27%; BHA 71.38 ± 3.81%; SHA 65.83 ± 2.66%). In addition, the highest biodegradation was observed in defects grafted with β-TCP (remaining particles: 19.22 ± 2.09%), while defects grafted with bovine HA (remaining particles: 24.14 ± 3.36%) and synthetic HA (remaining particles: 32.84 ± 2.57%) displayed a higher amount of residual bone grafting material with no significant differences. Excellent bone–particle contact was, however, noted for all test materials.

After 18 months, bone remodelling had further progressed, and only a slight increase in newly formed bone was noted. Defects grafted with β-TCP displayed the greatest amount of newly formed bone also after 18 months, closely followed by defects augmented with both HA-based materials, which displayed a similar amount of bone being present (β-TCP 75.45 ± 1.69%; BHA 73.94 ± 2.52%; SHA 72.01 ± 2.39%; *p* > 0.05). All bone substitute materials showed excellent bone–particle contact of almost 100% (TCP 98.86 ± 1.67%; SHA 98.76 ± 0.54%; BHA 98.25 ± 0.94%; *p* > 0.05). While TCP-grafted defects only displayed a residual particle area fraction of 9.49 ± 1.14%, a considerably higher amount of residual bone grafting material was present in defects grafted with synthetic HA (particle area fraction SHA 26.38 ± 1.75% vs. BHA 21.29 ± 1.99%, after 18 months, with only a minor increase in resorption having occurred between 12 and 18 months). The empty control defects were mainly filled by scar tissue, and limited bone formation had occurred at the defect margins after 18 months.

In summary, after 3 and 6 months, significantly greater new bone formation was observed in defects augmented with the β-TCP-based material. In addition, β-TCP exhibited the greatest biodegradability after 1, 3, 6, 12 and 18 months. SHA induced higher bone–particle contact at 2 weeks and greater bone formation (Table 6) and OC expression at 1 month compared to BHA (Table 7) at these early time points. Bone–particle contact was found to be comparable after 3, 6, 12 and 18 months for all grafting materials. After 12 and 18 months of implantation, excellent bony regeneration of the critical-size defects and bone remodelling were observed with all test materials, which displayed excellent bone-bonding behaviour. The TCP grafting material was mostly resorbed; only in a few areas were highly degraded particle residues still present, which displayed excellent bone-bonding behaviour including bone formation and migration of active osteoblasts into the pores of the degrading material (Table 8). With both HA-based bone grafting materials, only partial biodegradation of the particles had occurred by 18 months, i.e. the particles had not been fully resorbed. For SHA, bone formation within the degrading particles was observed as early as after 3 months, while BHA exhibited a slightly higher biodegradability at 12 months. Furthermore, neither inflammatory round cells nor foreign body cells nor any inflammatory tissue was noted in any areas.

## Discussion

Reconstruction of bone defects remains a prevalent and challenging topic in oral and maxillofacial surgery. With respect to bone augmentation of the maxilla and/or mandible, autologous bone grafts, such as iliac crest grafts, are still regarded as the ‘gold standard’ [53, 54]. However, bone harvesting is often associated with second-site surgery, which may lead to severe donor-site morbidity [32, 55, 56]. Therefore, the demand for alternative bone graft materials has steadily increased, resulting in the creation of numerous potential bone substitutes [57], among which CaP-based bone substitute materials such as HA and β-TCP are most frequently used [13–17]. In this study, we analyzed the bone regenerative capacity and bioactive characteristics of three different grafting materials: a novel synthetic HA (Osbone^®^), alloplastic β-TCP (Cerasorb^®^ M) and bovine-derived HA (Bio-Oss^®^). To this end, the expression of osteogenic markers in the various cell (fibroblasts, osteocytes, and osteoblasts) and matrix components (fibrous matrix, bone matrix and osteoid) of the osseous tissue, as well as new bone formation, degradation of the bone substitute material and bone–particle contact were assessed *ex vivo* after 14 days, 1 month, and 3, 6, 12 and 18 months of implantation. The grafting materials were implanted in CSBD in the ovine scapula, whose osseous microanatomy is similar to that of the mandible, and which is formed by intramembranous ossification just like the mandible. In addition, sheep models were implemented as a well-established large animal model for studying bone regeneration due to their similarity to human beings [58, 59].

Ideal bone substitutes are supposed to be osteoconductive and bioactive and thus promote osteogenesis [5] and ideally osteoinduction. The bioactive properties, which entail surface transformation and dissolution phenomena, are supposed to cause osteogenic progenitor cells to be attracted and attach to the bone substitute surface, differentiate into mature osteoblasts, subsequently form bone matrix at the surface and induce matrix mineralization. During this process, osteoblasts undergo differentiation, which can be divided into three stages: cellular proliferation, extracellular-matrix maturation and mineralization [60]. At each stage, characteristic osteogenic markers are expressed, whereby Col I characterizes the transition from the initial period of proliferation to extracellular-matrix maturation. ALP is expressed during the post-proliferative period of extracellular matrix maturation, and OC is highly expressed during extracellular matrix mineralization [61, 62]. Previous studies have proven the value of these markers regarding osteoblastic differentiation and osteogenesis *in vivo* [14, 60, 63] which were determined for the three tested bone graft substitutes and the empty defects in this study. In addition, earlier studies focusing on the time course of new bone formation concluded that the amount of bone formation and degradation of the augmentation material increases between 4 and 6 months after implantation [64]. The findings of our study suggest that even after 12 and 18 months, bone remodelling and particle degradation were still actively progressing for all grafting materials. In this regard, our study allowed us to characterize the bioactive behavior of alloplastic β-TCP-, synthetic HA- and bovine HA-based materials *in vivo* up to 18 months by analyzing their effect on osteogenic marker expression as well as the time course of the expression of various osteogenic markers.

Col I expression was already present in fibroblastic cells and fibrous matrix after 2 weeks in defects augmented with the three test materials and displayed its highest levels in osteoblasts after 3 months. This indicates an increase in extracellular matrix synthesis and osteoblast activity. After 1 month, OC expression in the bone matrix was significantly higher in defects grafted with TCP and SHA compared to BHA, which was accompanied by greater bone formation and is indicative of active-matrix mineralization. The highest OC marker expression in the cellular components was observed after 6 months for all tested materials, which is indicative of high extracellular matrix mineralization and bone remodelling activity. After 18 months, similar levels of OC expression were noted in defects grafted with both HA materials, which is in accordance with our findings of ongoing bone remodelling and particle degradation.

Although all three test materials are CaP-based bone grafting materials, they differ considerably in their chemical composition and fall into two different categories, i.e. HA- and β-TCP-based bone grafting materials. Apart from their chemical composition, there are other material properties, which play a fundamental role with respect to the osteoconductive and bone regenerative behaviour of bone grafting materials, such as granule size, macro and micro geometry, porosity, microstructural surface properties, surface roughness and surface area [65–67]. To create equal experimental conditions for the three test materials and in order to rule out an influence of this parameter on bone formation and regeneration, a granule size of 1000–2000 µm was chosen for all test materials in this study.

The superior bioactive behaviour of β-TCP noted in our study, especially after 3 and 6 months, may be due to its excellent surface properties, including calcium uptake at the material surface after contact with biological fluids [7] and support of adhesion and proliferation of osteoblasts [24–26], which enhances new bone formation [24, 27]. The results of previously published studies, which demonstrated a high level of degradation up to 95% for β-TCP [20, 68, 69], an optimal reactivity with the surrounding tissues [69] and increased vascularization [70, 71], are consistent with our findings. In general, β-TCP is known to possess a higher biodegradability than HA [22], which is in accordance with our findings at 1 month, and 3, 6, 12 and 18 months.

The osteoconductive properties of the clinically well-established bovine-derived HA-based material have been documented by numerous animal and clinical studies before and are consistent with our results [72–74]. The novel synthetic HA-based bone grafting material appears to be equal, if not superior, in terms of bioactivity and biocompatibility, since it induced greater bone–particle contact, higher bone formation and osteocalcin expression in the bone matrix at the early time points of 2 weeks and 1 month. Both HA-based grafting materials showed excellent bone regeneration of the critical-size defects in the sheep scapula over the 18-month study period, exhibiting remarkable osteoconductivity and biocompatibility. Both grafting materials facilitated excellent bone regeneration at 3 months and further bone remodelling at 6, 12 and 18 months, as well as high bone–particle contact, i.e. excellent bone-bonding behaviour and osseous integration without any fibrous encapsulation, foreign body, or inflammatory response; only subtle differences were noted. After 12 months, both HA-based materials revealed a higher percentage of residual particles and less new bone formation than the β-TCP-based material. Whereas defects grafted with the bovine-derived HA displayed slightly higher bone formation and biodegradation than the synthetic HA at 12 months, after 18 months, defects augmented with both HA-based materials displayed almost similar new bone formation, particle degradation and bone–particle contact. As such, biodegradation of synthetic HA was found to be slightly slower and lower than that of the bovine HA (and β-TCP), which met its design criteria, as it was designed for long-term stable contour augmentation and bone defects with higher mechanical strength requirements. The risk of disease transmission or immunological response can be fully ruled out by using a novel synthetic HA-based bone grafting material, while this is not completely possible with materials of bovine origin [43–45]. Therefore, the synthetic HA-based bone grafting material, with its comparable characteristics with respect to bioactivity, excellent bone regenerative capacity, bone-bonding behaviour and osseous integration, provides a genuine alternative to the bovine-derived HA-based material. The greater bone–particle contact at 2 weeks and bone formation and osteocalcin expression at 1 month point towards slightly higher bioactivity. In this context, it is noteworthy that after contact with body fluid upon implantation dissolution, reprecipitation, ion-exchange and serum protein adsorption events take place at the surface of bioactive calcium phosphate ceramics leading to surface transformation phenomena, which affect osteoblast attachment via integrin receptors (outside-in signalling) and subsequent intracellular signalling which modulates osteoblast differentiation and survival [75, 76]. Differences in these surface transformation events, in which fibronectin adsorption and conformational changes of the molecule induced by the underlying ceramic surface play a key role by exposing specific adhesive motifs within the fibronectin molecule for cell attachment via integrin receptors [76, 77], may have led to the differences in bone formation at the surface of the Osbone^®^ and Bio-Oss^®^ granules.

Troeltzsch et al. demonstrated that reliable and predictable augmentation of horizontal and vertical alveolar defects up to a width and height of ∼3.7 mm can be achieved with the use of particulate grafting materials, whereas larger defects require utilizing autogenous bone blocks [37], which is often accompanied by donor site morbidity. Therefore, the need for non-autogenous synthetic bone grafts suitable for the reconstruction of larger defects has become evident. Since an ideal non-autologous bone graft substitute needs to facilitate hard tissue and volume stability and maintain the shape of the augmented area in the long term, especially in the reconstruction of vertical and combined vertical and horizontal alveolar defects, detailed knowledge of the various characteristics of different bone grafting materials is utterly important. The data generated in our study provide a first insight into the bone regenerative capacity, bioactive properties, and biodegradability in terms of new bone formation, bone–particle contact, i.e. bone bonding behaviour, and osteogenic marker expression of a synthetic HA-based bone substitute as compared to established HA and TCP bone grafting materials at different time points up to 18 months after implantation. Consequently, they provide an excellent knowledge base for creating synthetic 3D-printed patient-specific bone graft substitute blocks as well as mouldable and injectable resorbable calcium phosphate-based bone grafting materials suitable for reconstructing these more challenging large horizontal and vertical bone defects. Respective research projects are currently underway [14, 78].

A previous study evaluated the formation of living bone at the place of implantation and compared, e.g. Osbone^®^, Cerasorb^®^ M, and a deproteinized bovine-derived cancellous bone HA (Endobone^®^) 12 months after implantation [79]. Radiologically, Osbone^®^ showed the strongest evidence for osteoconductivity after 12 months [79], which is in line with our clinical findings for the SHA. In contrast to our results for Cerasorb^®^ M, Kozakiewicz et al. reported only minor resorption 12 months after implantation [79], which might be explained by the merely radiological evaluation in this study without histological and immunohistochemical analysis.

The results of the current study furthermore point towards the excellent potential of combining SHA with TCP for alveolar ridge augmentation with the GBR technique and a staged approach to rapidly induce the formation of functional bone tissue in the central area of the alveolar ridge, in which the implant is placed so as to facilitate optimal bone-implant-contact upon osseointegration, while ensuring long-term stable contour augmentation by using SHA for the surface layer underneath the membrane.

## Conclusions

All three bone substitutes facilitated excellent defect regeneration after 6, 12 and 18 months without any inflammatory tissue response, with TCP showing the highest resorbability and superior bone formation after 1, 3, 6 and 12 months. All three tested materials displayed excellent osteoconductivity, bone-bonding behaviour and biocompatibility. With both HA materials, a considerable amount of residual grafting material was present after 12 and 18 months. The synthetic HA supported new bone formation, osteogenic marker expression and matrix mineralization and displayed excellent bone-bonding behaviour to an equal degree compared to the bovine-derived HA Furthermore, it induced greater bone-biomaterial contact, bone formation and OC expression in the bone matrix than BHA early on after 2 weeks and 1 month of implantation, respectively. As a result, synthetic HA can be regarded as a valuable alternative to the bovine-derived HA without the potential risk of disease transmission.

## Supplementary Material

rbae041_Supplementary_Data
