# Supplementary material for: Effect of a synthetic hydroxyapatite-based bone grafting material compared to established bone substitute materials on regeneration of critical-size bone defects in the ovine scapula
Source: Regen Biomater. 2024 Apr 24;11:rbae041. doi: 10.1093/rb/rbae041 (PMC11187503; doi:10.1093/rb/rbae041)
Supplement: rbae041_Supplementary_Data [file rbae041_supplementary_data.docx]

| Supplementary Table 2: Results of the Immunohistochemical Analysis and Scoring of Osteogenic Marker Expression of Osteocalcin | | | | | | | |
| --- | --- | --- | --- | --- | --- | --- | --- |
| Interval | Bone graft material | Osteoblast,  Mean ± SD | Osteocyte,  Mean ± SD | Fibroblastic cells,  Mean ± SD | Fibrous matrix,  Mean ± SD | Bone matrix,  Mean ± SD | Osteoid,  Mean ± SD |
| 2 weeks | Control | 0.00 ± 0.00 | 0.00 ± 0.00 | 1.0 ± 0.82 | 2.50 ± 0.58 | 0.00 ± 0.00 | 0.00 ± 0.00 |
|  | Bio-Oss® | 0.00 ± 0.00 | 0.00 ± 0.00 | 1.75 ± 1.50 | 2.25 ± 1.50 | 0.00 ± 0.00 | 0.00 ± 0.00 |
|  | Cerasorb® M | 0.00 ± 0.00 | 0.00 ± 0.00 | 3.25 ± 0.50 | 3.25 ± 0.50 | 0.00 ± 0.00 | 0.00 ± 0.00 |
|  | Osbone® | 0.00 ± 0.00 | 0.00 ± 0.00 | 2.50 ± 0.58 | 2.25 ± 0.50 | 0.00 ± 0.00 | 0.00 ± 0.00 |
| 1 month | Control | 0.00 ± 0.00 | 0.75 ± 1.50 | 2.00 ± 1.15 | 2.50 ± 1.00 | 0.25 ± 0.50 | 0.00 ± 0.00 |
|  | Bio-Oss® | 0.00 ± 0.00 | 2.25 ± 1.50 | 3.00 ± 0.00 | 3.75 ± 0.50 | 2.75 ± 1.89 | 0.00 ± 0.00 |
|  | Cerasorb® M | 0.00 ± 0.00 | 3.75 ± 0.50 | 3.00 ± 0.82 | 4.50 ± 0.58 | 4.00 ± 0.00 | 0.25 ± 0.50 |
|  | Osbone® | 0.00 ± 0.00 | 3.75 ± 0.50 | 3.25 ± 0.50 | 3.50 ± 0.58 | 4.00 ± 0.00 | 0.25 ± 0.50 |
| 3 months | Control | 1.00 ± 1.41 | 2.25 ± 1.50 | 3.00 ± 0.00 | 3.25 ± 0.50 | 1.75 ± 1.50 | 0.50 ± 0.58 |
|  | Bio-Oss® | 0.50 ± 0.58 | 3.00 ± 0.00 | 3.25 ± 0.50 | 3.50 ± 0.58 | 3.75 ± 0.50 | 1.00 ± 0.00 |
|  | Cerasorb® M | 0.75 ± 0.50 | 3.25 ± 0.50 | 2.75 ± 1.26 | 3.50 ± 0.58 | 3.75 ± 0.50 | 0.50 ± 0.58 |
|  | Osbone® | 0.75 ± 0.50 | 3.25 ± 0.50 | 2.75 ± 1.26 | 3.25 ± 0.50 | 3.50 ± 0.58 | 0.50 ± 0.58 |
| 6 months | Control | 0.75 ± 0.96 | 0.50 ± 1.00 | 3.00 ± 0.00 | 3.25 ± 0.50 | 3.25 ± 0.50 | 1.25 ± 1.26 |
|  | Bio-Oss® | 0.25 ± 0.50 | 4.00 ± 0.00 | 3.50 ± 0.58 | 3.25 ± 0.50 | 3.25 ± 0.50 | 0.50 ± 0.58 |
|  | Cerasorb® M | 0.50 ± 0.58 | 3.25 ± 0.50 | 3.50 ± 0.58 | 3.75 ± 0.50 | 4.25 ± 0.50 | 0.50 ± 0.58 |
|  | Osbone® | 0.50 ± 0.58 | 3.00 ± 0.00 | 3.25 ± 0.50 | 3.50 ± 0.58 | 4.25 ± 0.50 | 0.50 ± 1.00 |
| 12 months | Control | 0.50 ± 0.58 | 1.00 ± 0.82 | 4.00 ± 0.82 | 3.25 ± 0.50 | 3.75 ± 0.50 | 0.75 ± 0.96 |
|  | Bio-Oss® | 0.50 ± 0.58 | 3.00 ± 0.00 | 3.00 ± 0.00 | 3.25 ± 0.50 | 3.75 ± 0.50 | 0.50 ± 0.58 |
|  | Cerasorb® M | 0.75 ± 1.50 | 3.00 ± 0.00 | 2.50 ± 1.00 | 3.50 ± 0.58 | 4.00 ± 0.82 | 0.75 ± 1.50 |
|  | Osbone® | 1.25 ± 1.26 | 3.00 ± 0.00 | 2.50 ± 1.00 | 3.25 ± 0.50 | 3.75 ± 0.50 | 0.50 ± 1.00 |
| 18 months | Control | 1.75 ± 0.50 | 2.50 ± 1.29 | 2.75 ± 0.50 | 2.75 ± 0.50 | 2.75 ± 0.50 | 0.50 ± 0.58 |
|  | Bio-Oss® | 1.00 ± 0.00 | 3.50 ± 0.58 | 3.00 ± 0.00 | 2.50 ± 1.00 | 3.50 ± 0.58 | 1.50 ± 1.00 |
|  | Cerasorb® M | 0.75 ± 1.50 | 3.25 ± 0.50 | 3.75 ± 0.50 | 4.00 ± 0.00 | 4.00 ± 0.00 | 0.25 ± 0.50 |
|  | Osbone® | 0.75 ± 1.50 | 3.00 ± 0.00 | 3.50 ± 0.58 | 3.75 ± 0.50 | 3.75 ± 0.50 | 0.25 ± 0.50 |

| Supplementary Table 3: Results of the Immunohistochemical Analysis and Scoring of Osteogenic Marker Expression of Alkaline Phosphatase | | | | | | | |
| --- | --- | --- | --- | --- | --- | --- | --- |
| Interval | Bone graft material | Osteoblast,  Mean ± SD | Osteocyte,  Mean ± SD | Fibroblastic cells,  Mean ± SD | Fibrous matrix,  Mean ± SD | Bone matrix,  Mean ± SD | Osteoid,  Mean ± SD |
| 2 weeks | Control | 0.00 ± 0.00 | 0.00 ± 0.00 | 1.25 ± 0.50 | 2.75 ± 0.50 | 0.00 ± 0.00 | 0.00 ± 0.00 |
|  | Bio-Oss® | 0.00 ± 0.00 | 0.00 ± 0.00 | 2.00 ± 1.15 | 2.00 ± 1.15 | 0.00 ± 0.00 | 0.00 ± 0.00 |
|  | Cerasorb® M | 0.00 ± 0.00 | 0.00 ± 0.00 | 3.75 ± 0.50 | 3.50 ± 0.58 | 0.00 ± 0.00 | 0.00 ± 0.00 |
|  | Osbone® | 0.00 ± 0.00 | 0.00 ± 0.00 | 2.75 ± 0.50 | 3.00 ± 0.00 | 0.00 ± 0.00 | 0.00 ± 0.00 |
| 1 month | Control | 0.00 ± 0.00 | 1.75 ± 1.50 | 2.75 ± 0.50 | 3.00 ± 0.00 | 1.75 ± 1.50 | 0.25 ± 0.50 |
|  | Bio-Oss® | 0.25 ± 0.50 | 2.25 ± 1.50 | 3.00 ± 0.00 | 3.75 ± 0.50 | 2.25 ± 1.50 | 0.25 ± 0.50 |
|  | Cerasorb® M | 0.25 ± 0.50 | 3.25 ± 0.50 | 3.25 ± 0.50 | 4.25 ± 0.50 | 4.00 ± 0.00 | 0.50 ± 1.00 |
|  | Osbone® | 0.25 ± 0.50 | 3.00 ± 0.00 | 3.00 ± 0.00 | 3.75 ± 0.50 | 4.00 ± 0.00 | 0.25 ± 0.50 |
| 3 months | Control | 0.00 ± 0.00 | 3.00 ± 0.00 | 2.50 ± 1.00 | 3.25 ± 0.50 | 2.50 ± 1.00 | 0.00 ± 0.00 |
|  | Bio-Oss® | 1.00 ± 1.41 | 3.00 ± 0.00 | 3.25± 0.50 | 3.50 ± 0.58 | 3.25 ± 0.50 | 0.50 ± 0.58 |
|  | Cerasorb® M | 0.50 ± 0.58 | 3.25 ± 0.50 | 2.25 ± 0.96 | 3.25 ± 0.50 | 3.25 ± 0.50 | 0.25 ± 0.50 |
|  | Osbone® | 0.50 ± 0.58 | 3.25 ± 0.50 | 2.25 ± 0.96 | 3.00 ± 0.00 | 3.00 ± 0.00 | 0.25 ± 0.50 |
| 6 months | Control | 0.25 ± 0.50 | 3.00 ± 0.00 | 2.50 ± 0.58 | 3.00 ± 0.00 | 2.25 ± 0.96 | 0.50 ± 0.58 |
|  | Bio-Oss® | 0.50 ± 0.58 | 3.00 ± 0.00 | 3.25 ± 0.50 | 3.75 ± 0.50 | 3.00 ± 0.00 | 0.50 ± 0.58 |
|  | Cerasorb® M | 0.00 ± 0.00 | 3.00 ± 0.00 | 3.00 ± 0.00 | 3.75 ± 0.50 | 3.00 ± 0.00 | 0.00 ± 0.00 |
|  | Osbone® | 0.00 ± 0.00 | 3.00 ± 0.00 | 3.00 ± 0.00 | 3.25 ± 0.50 | 3.00 ± 0.00 | 0.00 ± 0.00 |
| 12 months | Control | 0.50 ± 0.58 | 3.25 ± 0.50 | 2.50 ± 0.58 | 3.75 ± 0.50 | 3.75 ± 0.50 | 0.50 ± 0.58 |
|  | Bio-Oss® | 0.25 ± 0.50 | 2.50 ± 1.00 | 3.00 ± 0.00 | 2.25 ± 1.50 | 2.75 ± 1.26 | 0.50 ± 0.58 |
|  | Cerasorb® M | 0.00 ± 0.00 | 3.00 ± 0.00 | 2.75 ± 0.00 | 3.25 ± 0.50 | 2.25 ± 0.50 | 0.00 ± 0.00 |
|  | Osbone® | 0.00 ± 0.00 | 3.25 ± 0.50 | 2.75 ± 0.50 | 3.25 ± 0.50 | 2.25 ± 0.50 | 0.00 ± 0.00 |
| 18 months | Control | 0.50 ± 0.58 | 3.25 ± 0.50 | 3.25 ± 0.50 | 2.75 ± 0.50 | 3.75 ± 0.50 | 0.25 ± 0.50 |
|  | Bio-Oss® | 0.25 ± 0.50 | 3.25 ± 0.50 | 3.00 ± 0.00 | 3.25 ± 0.50 | 2.50 ± 1.00 | 0.25 ± 0.50 |
|  | Cerasorb® M | 0.75 ± 0.50 | 3.00 ± 0.00 | 3.25 ± 0.50 | 3.25 ± 0.50 | 3.00 ± 0.82 | 0.00 ± 0.00 |
|  | Osbone® | 0.00 ± 0.00 | 3.00 ± 0.00 | 2.75 ± 0.50 | 3.25 ± 0.50 | 3.25 ± 0.50 | 0.00 ± 0.00 |

| Supplementary Table 4: Results of the Immunohistochemical Analysis and Scoring of Osteogenic Marker Expression of type I collagen | | | | | | | |
| --- | --- | --- | --- | --- | --- | --- | --- |
| Interval | Bone graft material | Osteoblast,  Mean ± SD | Osteocyte,  Mean ± SD | Fibroblastic cells,  Mean ± SD | Fibrous matrix,  Mean ± SD | Bone matrix,  Mean ± SD | Osteoid,  Mean ± SD |
| 2 weeks | Control | 0.00 ± 0.00 | 0.00 ± 0.00 | 1.50 ± 0.58 | 2.50 ± 0.58 | 0.00 ± 0.00 | 0.00 ± 0.00 |
|  | Bio-Oss® | 0.00 ± 0.00 | 0.00 ± 0.00 | 2.00 ± 1.15 | 2.75 ± 0.50 | 0.00 ± 0.00 | 0.00 ± 0.00 |
|  | Cerasorb® M | 0.00 ± 0.00 | 0.00 ± 0.00 | 4.00 ± 0.00 | 4.50 ± 0.58 | 0.00 ± 0.00 | 0.00 ± 0.00 |
|  | Osbone® | 0.00 ± 0.00 | 0.00 ± 0.00 | 3.00 ± 0.00 | 3.50 ± 0.58 | 0.00 ± 0.00 | 0.00 ± 0.00 |
| 1 month | Control | 0.00 ± 0.00 | 1.75 ± 1.50 | 3.00 ± 0.00 | 2.75 ± 0.50 | 1.50 ± 1.00 | 0.00 ± 0.00 |
|  | Bio-Oss® | 0.25 ± 0.50 | 3.00 ± 0.00 | 3.00 ± 0.00 | 4.00 ± 0.00 | 3.00 ± 0.00 | 0.25 ± 0.50 |
|  | Cerasorb® M | 0.00 ± 0.00 | 3.25 ± 0.50 | 3.00 ± 0.00 | 4.25 ± 0.50 | 3.25 ± 0.50 | 0.25 ± 0.50 |
|  | Osbone® | 0.00 ± 0.00 | 3.25 ± 0.50 | 3.00 ± 0.00 | 3.75 ± 0.50 | 3.00 ± 0.00 | 0.25 ± 0.50 |
| 3 months | Control | 0.00 ± 0.00 | 3.50 ± 0.58 | 2.50 ± 1.00 | 3.25 ± 0.50 | 3.50 ± 0.58 | 0.00 ± 0.00 |
|  | Bio-Oss® | 1.00 ± 0.00 | 2.75 ± 0.50 | 3.00 ± 0.00 | 3.50 ± 0.58 | 3.00 ± 0.00 | 0.75 ± 0.50 |
|  | Cerasorb® M | 0.50 ± 0.58 | 3.00 ± 0.00 | 3.00 ± 0.00 | 3.50 ± 0.58 | 3.25 ± 0.50 | 1.50 ± 1.73 |
|  | Osbone® | 0.50 ± 0.58 | 3.00 ± 0.00 | 3.00 ± 0.00 | 3.25 ± 0.50 | 3.25 ± 0.50 | 1.25 ± 1.26 |
| 6 months | Control | 0.75 ± 0.96 | 2.50 ± 1.00 | 2.50 ± 0.58 | 3.75 ± 0.50 | 3.50 ± 0.58 | 0.50 ± 0.58 |
|  | Bio-Oss® | 0.50 ± 0.58 | 3.00 ± 0.00 | 3.50 ± 0.58 | 3.75 ± 0.50 | 3.25 ± 0.50 | 1.75 ± 1.50 |
|  | Cerasorb® M | 0.00 ± 0.00 | 3.00 ± 0.00 | 2.00 ± 1.15 | 3.75 ± 0.50 | 3.50 ± 0.58 | 0.00 ± 0.00 |
|  | Osbone® | 0.00 ± 0.00 | 2.75 ± 0.50 | 1.75 ± 1.50 | 3.75 ± 0.50 | 3.50 ± 0.58 | 0.00 ± 0.00 |
| 12 months | Control | 0.50 ± 0.58 | 2.75 ± 0.50 | 3.00 ± 0.00 | 3.75 ± 0.50 | 3.00 ± 0.00 | 0.50 ± 0.58 |
|  | Bio-Oss® | 0.50 ± 0.58 | 3.25 ± 0.50 | 3.00 ± 0.00 | 3.50 ± 0.58 | 4.00 ± 0.00 | 0.50 ± 0.58 |
|  | Cerasorb® M | 0.00 ± 0.00 | 3.25 ± 0.50 | 2.75 ± 0.50 | 3.75 ± 0.50 | 3.75 ± 0.50 | 0.00 ± 0.00 |
|  | Osbone® | 0.00 ± 0.00 | 3.25 ± 0.50 | 2.75 ± 0.50 | 3.00 ± 0.00 | 3.75 ± 0.50 | 0.00 ± 0.00 |
| 18 months | Control | 0.50 ± 0.58 | 3.25 ± 0.50 | 3.25 ± 0.50 | 3.50 ± 0.58 | 3.50 ± 0.58 | 0.25 ± 0.50 |
|  | Bio-Oss® | 0.25 ± 0.50 | 3.25 ± 0.50 | 3.00 ± 0.00 | 3.25 ± 0.50 | 3.75 ± 0.50 | 0.00 ± 0.00 |
|  | Cerasorb® M | 0.00 ± 0.00 | 3.50 ± 0.58 | 3.75 ± 0.96 | 3.00 ± 1.41 | 3.25 ± 0.96 | 0.00 ± 0.00 |
|  | Osbone® | 0.00 ± 0.00 | 3.00 ± 0.00 | 3.75 ± 0.96 | 3.25 ± 0.96 | 3.25 ± 0.96 | 0.00 ± 0.00 |

| Supplementary Table 5: Results of the Immunohistochemical Analysis and Scoring of Osteogenic Marker Expression of bone sialoprotein | | | | | | | |
| --- | --- | --- | --- | --- | --- | --- | --- |
| Interval | Bone graft material | Osteoblast,  Mean ± SD | Osteocyte,  Mean ± SD | Fibroblastic cells,  Mean ± SD | Fibrous matrix,  Mean ± SD | Bone matrix,  Mean ± SD | Osteoid,  Mean ± SD |
| 2 weeks | Control | 0.00 ± 0.00 | 0.00 ± 0.00 | 1.50 ± 0.58 | 2.00 ± 0.00 | 0.00 ± 0.00 | 0.00 ± 0.00 |
|  | Bio-Oss® | 0.00 ± 0.00 | 0.00 ± 0.00 | 1.50 ± 1.00 | 2.25 ± 0.96 | 0.00 ± 0.00 | 0.00 ± 0.00 |
|  | Cerasorb® M | 0.00 ± 0.00 | 0.00 ± 0.00 | 2.50 ± 1.00 | 3.50 ± 0.58 | 0.00 ± 0.00 | 0.00 ± 0.00 |
|  | Osbone® | 0.00 ± 0.00 | 0.00 ± 0.00 | 1.75 ± 1.26 | 3.00 ± 0.00 | 0.00 ± 0.00 | 0.00 ± 0.00 |
| 1 month | Control | 0.00 ± 0.00 | 2.25 ± 1.50 | 2.50 ± 1.00 | 2.75 ± 1.26 | 1.75 ± 1.50 | 0.00 ± 0.00 |
|  | Bio-Oss® | 0.25 ± 0.50 | 3.00 ± 0.00 | 3.00 ± 0.00 | 4.00 ± 0.00 | 3.00 ± 0.00 | 0.25 ± 0.50 |
|  | Cerasorb® M | 0.00 ± 0.00 | 4.00 ± 0.00 | 3.75 ± 0.50 | 4.00 ± 0.00 | 3.50 ± 1.00 | 0.25 ± 0.50 |
|  | Osbone® | 0.00 ± 0.00 | 3.75 ± 0.50 | 3.50 ± 0.58 | 4.00 ± 0.00 | 3.00 ± 0.82 | 0.25 ± 0.50 |
| 3 months | Control | 0.00 ± 0.00 | 2.50 ± 1.00 | 2.00 ± 1.15 | 3.00 ± 0.00 | 2.50 ± 1.00 | 0.00 ± 0.00 |
|  | Bio-Oss® | 0.25 ± 0.50 | 3.00 ± 0.00 | 3.50 ± 0.58 | 4.00 ± 0.00 | 4.00 ± 0.00 | 0.25 ± 0.50 |
|  | Cerasorb® M | 0.50 ± 0.58 | 3.75 ± 0.50 | 3.00 ± 0.00 | 3.00 ± 0.00 | 3.75 ± 0.50 | 0.50 ± 0.58 |
|  | Osbone® | 0.50 ± 0.58 | 3.50 ± 0.58 | 3.00 ± 0.00 | 3.00 ± 0.00 | 3.25 ± 0.50 | 0.50 ± 0.58 |
| 6 months | Control | 0.25 ± 0.50 | 3.00 ± 0.00 | 2.50 ± 0.58 | 3.00 ± 0.00 | 2.75 ± 0.50 | 0.50 ± 0.58 |
|  | Bio-Oss® | 0.50 ± 0.58 | 3.00 ± 0.00 | 3.25 ± 0.05 | 3.50 ± 0.58 | 3.00 ± 0.00 | 1.00 ± 0.00 |
|  | Cerasorb® M | 0.50 ± 0.58 | 3.00 ± 0.00 | 3.00 ± 0.00 | 3.75 ± 0.50 | 3.75 ± 0.50 | 0.50 ± 0.58 |
|  | Osbone® | 1.25 ± 0.50 | 3.00 ± 0.00 | 3.00 ± 0.00 | 3.50 ± 0.58 | 3.50 ± 0.58 | 0.75 ± 0.50 |
| 12 months | Control | 0.50 ± 0.58 | 2.75 ± 0.50 | 3.00 ± 0.00 | 3.00 ± 0.00 | 3.00 ± 0.00 | 0.50 ± 0.58 |
|  | Bio-Oss® | 0.50 ± 0.58 | 3.50 ± 0.58 | 3.25 ± 0.50 | 3.75 ± 0.50 | 3.75 ± 0.50 | 0.50 ± 0.58 |
|  | Cerasorb® M | 0.00 ± 0.00 | 3.25 ± 0.50 | 3.50 ± 0.58 | 3.75 ± 0.50 | 3.50 ± 0.58 | 1.00 ± 1.15 |
|  | Osbone® | 0.00 ± 0.00 | 3.25 ± 0.50 | 3.00 ± 0.82 | 3.00 ± 0.82 | 3.00 ± 0.00 | 0.00 ± 0.00 |
| 18 months | Control | 0.50 ± 0.58 | 3.00 ± 0.82 | 3.00 ± 0.00 | 3.25 ± 0.50 | 3.00 ± 0.00 | 0.25 ± 0.50 |
|  | Bio-Oss® | 0.00 ± 0.00 | 3.50 ± 0.58 | 3.25 ± 0.50 | 3.50 ± 0.58 | 4.00 ± 0.00 | 0.00 ± 0.00 |
|  | Cerasorb® M | 0.00 ± 0.00 | 3.75 ± 0.50 | 3.00 ± 0.82 | 4.00 ± 0.00 | 3.75 ± 0.50 | 0.00 ± 0.00 |
|  | Osbone® | 0.00 ± 0.00 | 3.50 ± 0.58 | 2.75 ± 0.50 | 4.00 ± 0.00 | 3.50 ± 0.58 | 0.00 ± 0.00 |
